# The euro area’s pandemic recession: A DSGE-based interpretation^☆^

**DOI:** 10.1016/j.jedc.2022.104512

**Published:** 2022-10

**Authors:** Roberta Cardani, Olga Croitorov, Massimo Giovannini, Philipp Pfeiffer, Marco Ratto, Lukas Vogel

**Affiliations:** a Joint Research Centre, European Commission, TP581, 21027 Ispra (VA), Italy; b Directorate-General Economic and Financial Affairs, European Commission, Rue de la Loi 170, 1049 Brussels, Belgium; c Central Bank of Malta, Castille Place, Valletta VLT 1060, Malta

**Keywords:** COVID-19, Estimated DSGE model, Euro area, Recession, Forced savings

## Abstract

This paper augments the European Commission’s open-economy DSGE model (GM) with COVID-specific shocks (‘forced savings’, labour hoarding) and financially-constrained investors to account for the extreme volatility of private domestic demand and hours worked during COVID-19, and it estimates the model on euro area data for the period 1998q4–2021q4. It takes a pragmatic approach of adapting the workhorse model of a policy institution to COVID-19 data. ‘Forced savings’ are central to explain quarterly real GDP growth during the pandemic, complemented by contributions from foreign demand and trade, and the negative impact of persistently higher savings after the first wave. We provide extensive model validation, including a comparison to off-model evidence for COVID-related restrictions, and a comparison of different model specifications.

## 1 Introduction

This paper offers an economic interpretation of the COVID-19 crisis through the lens of an estimated structural macroeconomic model, focusing on the euro area (EA). The pandemic had multiple effects across different markets. Supply and demand constraints, associated with social distancing and lockdowns, led to a contraction of economic activity in contact-intensive sectors and the aggregate. Private consumption and trade collapsed in 2020. Liquidity squeezes and uncertainty caused tensions in financial markets. At the same time, governments implemented exceptional fiscal packages. The model disentangles the various factors (forced and precautionary savings, investment risk, trade exposure, labour hoarding, fiscal policy) and quantifies their importance. To the best of our knowledge, the paper is the first to characterise the macroeconomic consequences of COVID-19 through the lens of an estimated DSGE model for the EA. We use a pragmatic approach that allows to adapt workhorse DSGE models of policy institutions to economic analysis covering the COVID-19 period.

The analysis builds on the Global Multi-Country model (Albonico, Calais, Cardani, Croitorov, Ferroni, Giovannini, Hohberger, Pataracchia, Pericoli, Pfeiffer, Raciborski, Ratto, Roeger, Vogel, 2019, Giovannini, Hohberger, Kollmann, Ratto, Roeger, Vogel, 2019) and inspects shock decompositions (SDs) for economic activity at quarterly frequency. The fact that shocks during COVID-19 have been extremely large by historical standards poses a challenge for estimating models with stochastic disturbances. We overcome the problem by including (novel) COVID-specific shocks into the model. These shocks characterise ‘forced savings’ and large amounts of labour hoarding (the gap between hours paid and hours worked), mimicking short-time work schemes.

We are agnostic whether the COVID-19 recession is fundamentally a demand- (‘scared consumers’) or supply-side (mandated closures) event. Demand and supply shocks are usually identified by the output-inflation co-movement. The transitory shocks necessary to fit the volatile quarterly profile of economic activity, particularly in 2020, have little impact on inflation in a model with (sufficiently) sticky prices, however.

The benchmark version of the estimation exploits the fact that the timing of COVID-19 is known, i.e. no shock before 2020, similarly to Lenza and Primiceri (2022). Technically, the approach translates into a model where a subset of shocks displays exogenous deterministic heteroskedasticity. Lifting this restriction reproduces a similar shock profile, however. A heteroskedastic filter allows the standard errors of the COVID-specific shocks to vary between pre-COVID and COVID periods, which finally also allows for an estimation of the model, including 2020-21 data.

The estimated model identifies domestic saving shocks as key drivers of the EA economy’s quarterly GDP growth profile in 2020-21. Early in the pandemic, this relates to transitory ‘forced savings’, but later increasingly to more persistent saving shocks, reflecting precautionary motives or the fact that restrictions became more entrenched (and foreseeable) the longer the pandemic persisted. Comparison to a model variant without COVID-specific shocks demonstrates the gains from the extensions in terms of model fit. The non-persistent savings shock captures well the first wave of the pandemic in 2020, whereas a more persistent (moving average) savings shock becomes subsequently more important. The model also features liquidity-constrained firms whose investment is constrained by (falling) gross operating surpluses in response to declining consumption and export demand.

The paper proceeds in the following steps: Section 2 sketches related literature on the economic impact of COVID-19 to place the paper and its purpose in context. Section 3 presents stylised facts to characterise the EA macroeconomy during the pandemic. Section 4 outlines the main elements of the model. Section 5 describes the econometric approach and reports parameter estimates. The propagation of COVID-specific and other important shocks is analysed in more detail in Section 6. Section 7 provides a quantitative assessment of the main drivers of EA growth and inflation at quarterly frequency during COVID-19 and includes a comparison with the Global Financial Crisis (GFC) of 2008-09 as well as an analysis of growth forecast error in 2020-21. Section 8 provides additional evidence for the COVID-specific shocks in the model. Section 9 assesses the model fit in comparison to alternative specifications of the savings shock. Section 10 summarises the findings and concludes.

## 2 Related literature

This paper contributes to the literature on the macroeconomic impact of the COVID-19 pandemic. It belongs to the contributions that adapt workhorse DSGE models to the COVID shock. Within this literature, Chen et al. (2020) show that the New York Fed DSGE model, augmented by COVID-specific supply and demand shocks, interprets the pandemic recession as a demand shock to the US economy. Corrado et al. (2021) estimate a two-sector New Keynesian model on US data to analyse demand and supply contributions. Their model identifies strong negative demand shocks in both sectors, a large labour supply shock to the not-directly-affected sector, and a large labour productivity shock in the sector directly affected by the pandemic. Kollmann (2021) uses a stylised New Keynesian model at annual frequency and argues that the aggregate supply shock has been the main driver of the sharp GDP contraction in the EA in the pandemic, whereas both aggregate demand and supply shocks are necessary to fit the relative stability of inflation.^1^

COVID-19 has sparked a much richer literature on ‘non-standard’ structural macro models on which we build for our ‘reduced-form’ modelling of the pandemic and the interpretation of our results. One strand of this literature has merged epidemiology and macro models to understand the dynamic interaction between the pandemic, containment policies, and economic activity. Prominent examples are Eichenbaum et al. (2021), Eichenbaum et al. (2022), Jones et al. (2021), and Moser and Yared (2021). Bodenstein et al. (2022) consider a unidirectional link, where health dynamics affect the labour supply in the economy. Our standard DSGE model does not include an epidemiology block, but we build on this literature to understand the waviness of pandemics and the rationale for containment policies.^2^

Another strand of papers develops multi-sector models and highlights the importance of sector linkages and spillover. Guerrieri et al. (2022) show in a stylised two-sector model how incomplete markets and complementarity in demand for sectoral output give rise to ‘Keynesian’ supply shocks, where a negative supply shock in the restricted sector (e.g. contact-intensive services) generates negative demand externalities that lead to lower activity also in the unrestricted sector. A targeted fiscal response in this situation uses transfers to stabilise the income of workers in the restricted sector instead of stimulating production in the restricted sector through additional public sector demand. Similarly, Woodford (2022) illustrates the working of demand externalities in a multi-sector model with nominal (price) rigidities but based on the network structure of demand and payments, and equally makes a case for fiscal transfers as a stabilisation tool in the presence of borrowing constraints. Baqaee and Farhi (2022) build a multi-sector model with rich input-output linkages and analyse the impact of sector-specific demand and supply shocks in the pandemic on output, employment, and inflation. In light of the asymmetric nature of the shocks, some sectors are tight (supply-constrained), while others are slack (demand-constrained). Complementarities in production reduce the effectiveness of aggregate demand stimulus, providing a case for income insurance (such as government transfers) rather than more government purchases under pandemic conditions. We use a one-sector macro model instead but borrow the difficulty to distinguish between demand and supply shocks in the aggregate in the case of COVID-19 and the explanation for the prominence of government transfers (e.g. via short-term work schemes) as a stabilisation tool.

The paper, finally, links to the literature that addresses the treatment of extreme economic fluctuations in econometric analysis. Several authors have stressed that the time-series properties of macro data are severely affected by the observations during COVID-19, posing challenges to forecasting and inference. In particular, the extreme fluctuations translate into unstable parameter estimates, a deterioration of the pre-COVID-19 fit and poor forecasting ability of existing models, including observations from the pandemic.

The literature has suggested alternative solutions to overcome this challenge. Generally speaking, existing studies treat extreme observations as outliers, add additional shocks to the model, modify the assumptions on the shock covariance matrix, or relax the Gaussian hypothesis and allow for fat tail distributions. Schorfheide and Song (2021), e.g., estimate a mixed-frequency VAR to generate macroeconomic forecasts for the US during the COVID-19 pandemic and exclude crisis observations from the estimation sample. Ng (2021) interprets COVID-19 as an addition of new shocks to the economy and uses COVID-19 indicators from epidemiology data as exogenous controls to ‘de-covid’ the data prior to VAR estimation. Lenza and Primiceri (2022) estimate a VAR for the US at monthly frequency and explicitly model the change in shock volatility with priors on volatility scaling factors for March-May 2020 and on a decay parameter that determines the speed of convergence of the covariance towards the pre-COVID-19 values. Carriero et al. (2022) conduct a Bayesian VAR analysis and show that allowing for stochastic volatility together with volatility outliers and, possibly, fat-tailed errors produces estimates and forecasts that are less sensitive to COVID-19 realisations.

In the context of the DSGE literature, Chen et al. (2020) add partly anticipated i.i.d COVID-19 shocks to the discount factor, labour supply and productivity to capture the large disturbances in 2020-21 and the fact that agents could partly anticipate the wave-like dynamics after 2020q1. Corrado et al. (2021) instead estimate a non-linear non-Gaussian DSGE model. The authors show considerable computational gains of their non-linear filter over the particle filter, but it remains an open question whether estimation remains feasible for models with a data set larger than theirs. Our approach is to accept a compromise, in which we may neglect non-linearity beyond the occasionally binding effective lower bound (ELB), but with the advantage of being able to approach the empirical intricacies presented by COVID-19 within the class of medium-to-large scale models typically used in policy institutions. Our benchmark introduces COVID-specific shocks, which can also be characterised as shocks with deterministic exogenous heteroskedasticity. However, removing the heteroskedasticity assumption shows that estimation recovers a very similar shock profile over the sample horizon. In sum, our contribution - similar to Lenza and Primiceri (2022) in a VAR context and Chen et al. (2020) in a DSGE framework for the US - is to propose a pragmatic approach to address the empirical challenges associated with COVID-19 data in an otherwise established pre-COVID DSGE model for the EA.

## 3 Stylised facts

**Fig. 1:**
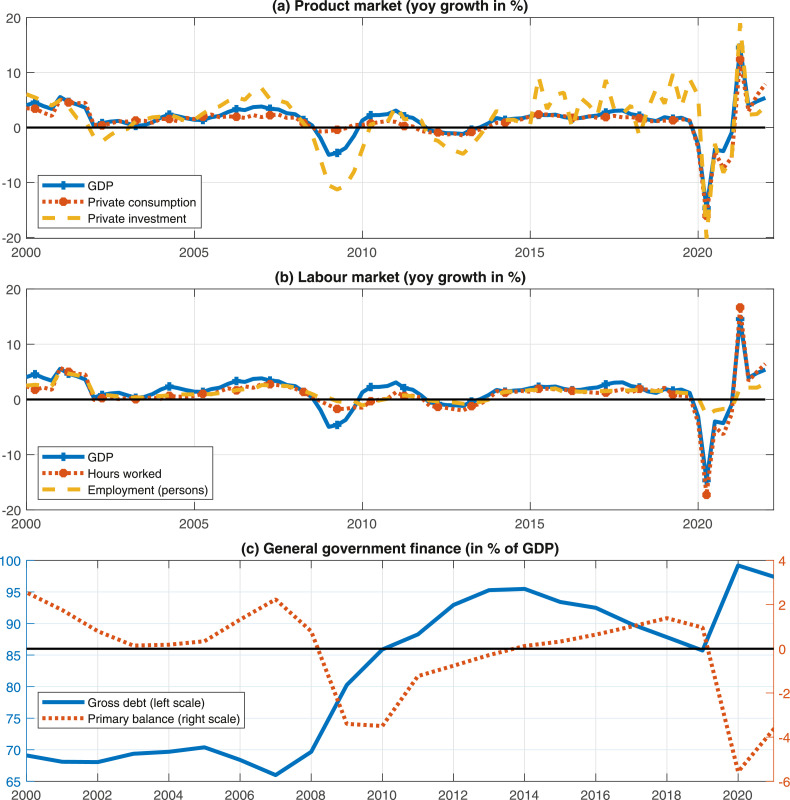
Stylised facts of the COVID recession in the EA economy. *Notes:* Consumption, investment and GDP are in constant prices, i.e. real terms. Data in Panels (a) and (b) is quarterly (year-on-year growth rates), whereas data in Panel (c) is annual. *Sources:* AMECO and Eurostat.

Figure 1 summarises a number of stylised facts about the macroeconomic impact of the COVID-19 pandemic in the EA. First, the size of the contraction of economic activity, at a quarterly frequency, in 2020q2 is unprecedented in recent history, including the GFC (Panel a).

Second, while real GDP in 2020q3-4 was still lower than in 2019q3-4, the EA economy recovered quickly from the low point in 2020q2, implying strong quarter-on-quarter growth in 2020q3. The V-shape in 2020 contrasts with the more persistent U-shape of the 2008-09 recession. Mirroring the 2020 contraction, EA year-on-year growth recorded an unprecedented in recent history recovery in 2021, notably in 2021q2 compared to 2020q2, with an inverted V-shape.

Third, private consumption and investment in the EA fell in tandem and to a similar extent in 2020, and they have recovered at a similar speed since. These dynamics contrast with investment growth being more volatile than consumption growth in ‘normal’ times and with consumption falling less than investment also during the GFC.

Fourth, the number of persons employed has remained rather stable compared to the dramatic contraction of hours worked in the pandemic in line with real GDP (Panel b). The difference contrasts with the close co-movement between hours and persons during the preceding two decades. The wedge between hours worked and employment in persons points to labour hoarding, notably in the form of short-time work arrangements, during the pandemic.

Finally, large fiscal packages during the COVID-19 crisis, with a focus on stabilising disposable income, and lower tax revenue have led to a sharp deterioration in the government’s primary balance, from 1% of GDP in 2019 to −6% in 2020 and −4% in 2021, and an increase in government debt to GDP by almost 15 percentage points (Panel c). Our estimated EA model with the COVID-specific model extensions has to account for these observations.

## 4 Model economy

The model outlined in this section is a standard quantitative macro model enriched to capture pandemic-specific features.^3^ It features two regions, namely the EA and the rest of the world (RoW). The superscript ${}^{*}$ denotes RoW variables and parameters.

The EA economy consists of households, a continuum of intermediate goods producers in monopolistic competition, final goods firms in perfect competition, import and export sectors, and a government. The EA final goods producers use EA intermediate goods, imported commodities (‘industrial supplies’) and imported manufactured goods as inputs. Wages are set by trade unions and sticky. The RoW block has a simpler structure than the EA economy, and it is the only supplier of commodities. Trade in goods and a financial asset link the EA with the RoW. To provide an empirically plausible account of the macroeconomic environment at quarterly frequency, the model includes nominal and real rigidities. Unless stated otherwise, all exogenous random variables follow autoregressive processes of order 1. Time is discrete and indexed by t.

### 4.1 Production

*Output and value added*. Perfectly competitive firms produce output ($O_{t}$) by combining domestic value added ($Y_{t}$) and imported industrial supplies ($IS_{t}$) in a CES production function

(1) $$O_{t}=[{\left(1-s_{t}^{IS}\right)}^{\frac{1}{\sigma^{o}}}{\left(Y_{t}\right)}^{\frac{\sigma^{o}-1}{\sigma^{o}}}+{\left(s_{t}^{IS}\right)}^{\frac{1}{\sigma^{o}}}{\left(IS_{t}\right)}^{\frac{\sigma^{o}-1}{\sigma^{o}}}]{}^{\frac{\sigma^{o}}{\sigma^{o}-1}},$$

where $s_{t}^{IS}$ is the input share of industrial supplies. This share is stochastic and captures fluctuations in the IS intensity of production.^4^ $\sigma^{o}>0$ is the elasticity of substitution between the two factors. Profit-maximisation implies

(2) $$Y_{t}=\left(1-s_{t}^{IS}\right){\left(\frac{P_{t}}{P_{t}^{O}}\right)}^{-\sigma^{o}}O_{t},$$

and

(3) $$IS_{t}=s_{t}^{IS}{\left(\frac{P_{t}^{IS}}{P_{t}^{O}}\right)}^{-\sigma^{o}}O_{t},$$

where $P_{t}$ and $P_{t}^{IS}$ are the price of value-added and the price of industrial supplies, respectively. The price of output equals marginal costs

(4) $$P_{t}^{O}=[\left(1-s_{t}^{IS}\right){\left(P_{t}\right)}^{\sigma^{o}-1}+s_{t}^{IS}{\left(P_{t}^{IS}\right)}^{\sigma^{o}-1}]{}^{\frac{1}{1-\sigma^{o}}}.$$

The commodities are imported from the RoW subject to an excise duty $\tau^{IS}$, so that

(5) $$P_{t}^{IS}=E_{t}P_{t}^{IS,*}+\tau^{IS}P_{t}^{w},$$

where $E_{t}$ is the nominal exchange rate between EA and RoW, and $P_{t}^{w}$ is the global GDP deflator.^5^

Value added $Y_{t}$ aggregates EA intermediate goods

(6) $$Y_{t}=[\int_{0}^{1}Y_{i,t}^{\frac{\sigma^{y}-1}{\sigma^{y}}}di]{}^{\frac{\sigma^{y}}{\sigma^{y}-1}},$$

where $Y_{i,t}$ denotes intermediate good i∈[0,1]. $\sigma^{y}>0$ is the elasticity of substitution between the varieties $Y_{i,t}$. The production function for good i is

(7) $$Y_{i,t}={\left(A_{t}^{Y}N_{i,t}\right)}^{\alpha }{\left(cu_{i,t}K_{i,t-1}\right)}^{1-\alpha }-A_{t}^{Y}\Phi ,$$

where $A_{t}^{Y}$ is an exogenous stochastic technology level, subject to trend and level shocks. $N_{i,t}$, $K_{i,t-1}$, and $cu_{i,t}$ are firm i’s labour input, capital stock, and endogenous capacity utilisation, respectively. Φ are fixed costs. Appendix A reports additional details. Gross investment $I_{i,t}$ drives the law of motion for capital $K_{i,t}=K_{i,t-1}\left(1-\delta \right)+I_{i,t}$, with 0<δ<1.

*Labour hoarding*. In light of the restrictions on work during COVID-19, we augment the model with a transitory ‘labour hoarding’ shock. This labour demand shock captures short-time work arrangements, i.e. employees working less while remaining employed. By changing the labour intensity of production at the intensive margin, without hiring or firing costs for firms, this shock introduces a wedge between effective hours worked (production function) and hours paid (wage income) in the model. Labour hoarding enters as one-off shock to hours, $\varepsilon_{t}^{tN}$, and the sum of the shock and hours effectively worked, $N_{i,t}$, equals hours paid, $N_{i,t}^{paid}$. Period t dividends are

(8) $$D_{i,t}=P_{i,t}Y_{i,t}-W_{t}\left(N_{i,t}+\varepsilon_{t}^{tN}\right)-P_{t}^{I}I_{i,t}-\Gamma_{i,t}.$$

$W_{t}$ and $P_{t}^{I}$ are the nominal wage rate and the price of investment goods, respectively. $\Gamma_{i,t}$ collects quadratic price and factor adjustment costs. Each intermediate goods firm i sets the good’s price $P_{i,t}$ in a monopolistically competitive market, subject to Rotemberg (1982) price adjustment costs and the demand function $Y_{i,t}={\left(\frac{P_{i,t}}{P_{t}}\right)}^{-\sigma^{y}}Y_{t}$. The share (1−sfp) of firms indexes prices to past inflation. Appendix A presents the equilibrium conditions for the firm sector.

*Liquidity-constrained investment*. Following Pfeiffer et al. (2020), we assume that a share of intermediate goods firms faces a temporary liquidity constraint. This time-varying share, $sli_{t}$, depends on the aggregate gross operating surplus, following the linear relation

(9) $$sli_{t}=a_{0}-a_{1}\left(\frac{GOS_{t}}{K_{t-1}}\frac{P_{t}}{P_{t}^{I}}\right),$$

with parameters $a_{0}$ and $a_{1}$. We define the firm-specific gross operating surplus as $\mathrm{GO}S_{i,t}\equiv Y_{i,t}-W_{t}/P_{i,t}\left(N_{i,t}+\varepsilon_{t}^{\mathrm{tN}}\right)$. We assume that for liquidity-constrained firms $i\in [0,\mathrm{sl}i_{t}]$, the net investment rate follows

(10) $$\frac{I_{t}}{K_{t}}-\delta =H\left(\frac{I_{i,t}}{K_{i,t}}\right)\equiv \zeta_{1}\left(\frac{\mathrm{GO}S_{i,t}}{K_{i,t-1}}\frac{P_{t}}{P_{t}^{I}}\right)-\zeta_{0},$$

where parameters $\zeta_{0}$ and $\zeta_{1}$ govern the strength of the liquidity constraint. Pfeiffer et al. (2020) sketch a microfoundation of this functional form based on a model of loan restrictions. Our parametrisation implies that a decline of available funds reduces investment demand in the presence of adverse demand or supply shocks. As investment decisions of unconstrained firms ($i\in (\mathrm{sl}i_{t},1]$) follow a standard Q-equation, denoted $F\left(Q_{i,t}\right)$, total private investment is given by

(11) $$\frac{I_{t}}{K_{t}}-\delta =\int_{0}^{\mathrm{sl}i_{t}}H\left(\frac{I_{i,t}}{K_{i,t}}\right)\;\mathrm{di}+\int_{\mathrm{sl}i_{t}}^{1}F\left(Q_{i,t}\right)\;\mathrm{di}.$$

### 4.2 Trade

Let $Z_{t}\in \left\{C_{t},G_{t},I_{t},IG_{t},X_{t}\right\}$ be the final demand by households and the government, private and government investors, and exporters, respectively. Perfectly competitive firms assemble $Z_{t}$, using domestic output and imported inputs ($M_{t}^{Z}$) in a CES production function:

(12) $$Z_{t}=A_{t}^{p,Z}[{\left(1-s_{t}^{M,Z}\right)}^{\frac{1}{\sigma^{z}}}{\left(O_{t}^{Z}\right)}^{\frac{\sigma^{z}-1}{\sigma^{z}}}+{\left(s_{t}^{M,Z}\right)}^{\frac{1}{\sigma^{z}}}{\left(M_{t}^{Z}\right)}^{\frac{\sigma^{z}-1}{\sigma^{z}}}]{}^{\frac{\sigma^{z}}{\sigma^{z}-1}},$$

where $A_{t}^{p,Z}$ denotes a productivity shock in sector Z. $0<s_{t}^{M,Z}<1$ is the sector-specific stochastic import share.^6^ $\sigma^{z}>0$ is the elasticity of substitution between domestic output and imported ones. It is assumed to be common across all final demand components. The resulting demand functions for domestic and imported components are

(13) $$O_{t}^{Z}={\left(A_{t}^{p,Z}\right)}^{\sigma^{z}-1}\left(1-s_{t}^{M,Z}\right){\left(\frac{P_{t}^{O}}{P_{t}^{Z}}\right)}^{-\sigma^{z}}Z_{t}$$

and

(14) $$M_{t}^{Z}={\left(A_{t}^{p,Z}\right)}^{\sigma^{z}-1}s_{t}^{M,Z}{\left(\frac{P_{t}^{M}}{P_{t}^{Z}}\right)}^{-\sigma^{z}}Z_{t},$$

where the price deflator associated with $Z_{t}$ is

(15) $$P_{t}^{Z}={\left(A_{t}^{p,Z}\right)}^{-1}[\left(1-s_{t}^{M,Z}\right){\left(P_{t}^{O}\right)}^{1-\sigma^{z}}+s_{t}^{M,Z}{\left(P_{t}^{M}\right)}^{1-\sigma^{z}}]{}^{\frac{1}{1-\sigma^{z}}},$$

and $P_{t}^{M}=E_{t}P_{t}^{X,*}.$

### 4.3 Households

*Savers*. Two groups of representative households consume and provide labour to intermediate good producers. A share $\omega^{s}$ of households are savers (s), who own domestic firms and participate in financial markets (saving and borrowing). Savers choose consumption $C_{j,t}^{s}$ and assets $B_{j,t}^{Q}$ to maximise welfare

(16) $$E_{0}\sum_{t=0}^{\infty }\beta^{t}\xi_{t}\left\{\frac{{\left(C_{j,t}^{s}-\varepsilon_{t}^{\mathrm{tC}}-h\left(C_{t-1}^{s}-\varepsilon_{t-1}^{\mathrm{tC}}\right)\right)}^{1-\theta }}{1-\theta }-\omega_{t}^{N}\frac{{\left(N_{j,t}^{s}\right)}^{1+\theta^{N}}}{1+\theta^{N}}-\frac{{\bar{\lambda^{s}}}_{t}}{P_{t}^{C}}\sum_{Q}B_{j,t-1}^{Q}\left(\alpha^{Q}-\varepsilon_{t-1}^{Q}\right)\right\},$$

subject to the budget constraint:

(17) $$P_{t}^{C}C_{j,t}^{s}+\sum_{Q}B_{j,t}^{Q}=W_{t}\left(N_{j,t}^{s}+\varepsilon_{t}^{\mathrm{tN}}\right)+D_{t}+\sum_{Q}R_{t-1}^{Q}B_{j,t-1}^{Q}+T_{j,t}^{s},$$

where $0<\theta ,\theta^{N}$. h governs the importance of external consumption habits. $\xi_{t}$ captures stochastic disturbances to the discount factor β.^7^$\omega_{t}^{N}$ is a stochastic labour disutility term.^8^ $T_{j,t}^{s}$ summarises the taxes and transfers, which are detailed in the Appendix.

A novel aspect of the model compared to the standard GM specification in Albonico et al. (2019) are the non-persistent ‘forced savings’ shocks, $\varepsilon_{t}^{tC}$, that constrain consumption outside of habit persistence and are zero before 2020 in the benchmark version of the model.^9^

The portfolio $\sum_{Q}B_{j,t+1}^{Q}$ with gross nominal returns $R_{t}^{Q}$ consists of risk-free private domestic bonds (rf), government bonds (g), an internationally traded bond (bw), and domestic corporate shares (S).^10^ We incorporate assets in the utility function with asset-specific risk premia shocks $\varepsilon_{t}^{Q}$ with Q∈{bw,g,S}.^11^ Asset-specific intercepts, $\alpha^{Q}$, capture the steady-state risk premia, except for risk-free bonds. Fisher (2015) interprets an increase in $\varepsilon_{t}^{Q}$ as a wedge between the return on risky assets and safe bonds.^12^

*Hand-to-mouth*. The remaining households $\left(1-\omega^{s}\right)$ with the label (c) are ‘hand-to-mouth’ and face a liquidity constraint (or a zero-borrowing constraint) in that they simply consume their current net disposable income, which consists of wages and transfers minus taxes paid. The constraint binds in every period, except during COVID-19, where even these households accumulate ‘forced savings’ that will be spent gradually upon exit from the pandemic. Therefore, the budget constraint of liquidity-constrained households is:

(18) $$P_{t}^{C}C_{j,t}^{c}=W_{t}\left(N_{j,t}^{c}+\varepsilon_{t}^{tN}\right)+T_{j,t}^{c}+P_{t}^{C}(\varepsilon_{t}^{tC}-\frac{1}{6}\sum_{i=8}^{13}\varepsilon_{t-i}^{tC}).$$

Total consumption and hours worked by EA households are $C_{t}=\left(1-\omega^{s}\right)C_{t}^{c}+\omega^{s}C_{t}^{s}$ and $N_{t}=\left(1-\omega^{s}\right)N_{t}^{c}+\omega^{s}N_{t}^{s}$, respectively.

### 4.4 Wage setting

Wage setting is standard along the lines of Ratto et al. (2009) and Kollmann et al. (2016). A monopolistic EA trade union ‘differentiates’ homogeneous EA labour hours provided by the two domestic households into imperfectly substitutable labour services. The union then offers these services to local intermediate goods firms. The labour input $N_{i,t}$ in the production functions of those firms is a CES aggregate of differentiated labour services. The union sets wage rates at a markup over the marginal rate of substitution between leisure and consumption. The wage markup is inversely related to the substitutability between labour varieties in intermediate good production. We introduce nominal wage rigidity through quadratic wage adjustment costs, captured by the parameter $\gamma^{w}$. In addition, the parameter $\gamma^{wr}$ adds real wage rigidity as in Blanchard and Galí (2007) and Coenen and Straub (2005). A share (1−sfw) of unions sets wages indexed to past inflation. The real wage follows

(19) $$(mrs_{t}-\mu_{t}^{w}){}^{1-\gamma^{wr}}(\frac{W_{t-1}}{P_{t-1}^{C}}){}^{\gamma^{wr}}=\frac{W_{t}}{P_{t}^{C}},$$

where $mrs_{t}$ is the average (across the two household types) marginal rate of substitution between consumption and leisure, which weighs both households by their population share. $\mu_{t}^{w}$ denotes the gross wage markup, which fluctuates due to backward-looking wage setting and nominal frictions (see Appendix A). Employment is the same across both types of households.

### 4.5 Monetary and fiscal policy

*Monetary policy*. The EA monetary policy follows a Taylor (1993) rule, subject to an occasionally binding ELB constraint. The target interest rate $i_{t}^{not}$ responds sluggishly to deviations of inflation and the output gap ($Y_{t}^{gap}$) from their respective target levels

(20) $$i_{t}^{not}-\bar{i}=\rho^{i}\left(i_{t-1}-\bar{i}\right)+\left(1-\rho^{i}\right)[\frac{\eta^{i\pi }}{4}(\pi_{t}^{C,QA}-{\bar{\pi }}^{C,QA})+\frac{\eta^{iy}}{4}Y_{t}^{gap}],$$

where $\bar{i}$ denotes the steady-state nominal interest rate. $\pi_{t}^{C,QA}$ denotes quarterly annualised inflation, and ${\bar{\pi }}^{C,QA}$ is its steady-state value.^13^Variable $i_{t}$ is the actual or effective short-term interest rate. $\rho^{i}$, $\eta^{i\pi }$, $\eta^{iy}$ govern interest rate inertia and the response to annualised inflation and the output gap, respectively. The output gap equals the (log) difference between actual and potential output. Potential output at date t is the output level that would prevail if the labour input equalled the amount of hours worked in the absence of nominal wage rigidity as in Galí (2011), the capital stock was utilised at full capacity, and the TFP equalled its trend component.

The effective policy rate $i_{t}$ equals the target nominal short-term rate only when the latter is above the ELB (which we set at zero). The effective policy rate hence satisfies

(21) $$i_{t}=\max\left\{i_{t}^{not},0\right\}+\varepsilon_{t}^{i},$$

where $\varepsilon_{t}^{i}$ is a white noise monetary policy shock.

*Fiscal policy*. The fiscal authority raises constant linear taxes on consumption, wage income and corporate profits and collects a commodity import duty and lump-sum taxes (introduced to close the government budget) to finance its consumptive purchases, investments, and transfers.^14^ The individual government expenditure components follow feedback rules.

### 4.6 Resource constraint

The resource constraint of the EA economy is

(22) $$P_{t}Y_{t}+\tau^{IS}P_{t}^{w}IS_{t}=P_{t}^{C}C_{t}+P_{t}^{I}I_{t}+P_{t}^{IG}IG_{t}+P_{t}^{G}G_{t}+TB_{t},$$

where $P_{t}^{IG}$ and $P_{t}^{G}$ denote the prices of government investment and consumption goods, respectively, and

(23) $$TB_{t}\equiv P_{t}^{X}X_{t}-E_{t}P_{t}^{\mathrm{IS},*}IS_{t}-\sum_{z}P_{t}^{M}M_{t}^{Z}$$

defines the EA trade balance as the difference between exports and imports in value terms.

### 4.7 Rest of the world

The RoW block follows a simplified structure, excluding liquidity-constrained households and fiscal policy. Perfectly competitive final-goods firms (that bundle the final consumption and investment good $C_{t}^{*}\;\text{and}\;I_{t}^{*}$, respectively, and the RoW (non-commodity) export good $X_{t}^{*}$) follow a structure that is analogous to the determination of EA aggregate demand components outlined in Section 4.2, i.e. a bundling of domestic and imported output.^15^ The model includes a pricing-to-market feature (EA exporters follow a similar structure). In line with Itskhoki and Mukhin (2021), we assume that exporters partially target the destination output price. Hence, the desired export price depends on the marginal cost $\left(mc_{t}^{X,*}\right)$ and the destination output price, weighted by its respective elasticity. We also allow for price shock  $\varepsilon_{t}^{X^{*}}.$ Thus,

(24) $$P_{t}^{X^{*}}={\left(mc_{t}^{X,*}\right)}^{1-\alpha^{x}}{\left(\frac{1}{E_{t}}P_{t}^{0}\right)}^{\alpha^{x}}\exp\left(\varepsilon_{t}^{X^{*}}\right).$$

Monopolistically competitive RoW intermediates goods firms use a Cobb-Douglas technology as in Eq. (7) to produce domestic (non-commodity) output. The price setting for non-oil output follows a New Keynesian Phillips curve with a cost-push shock. The RoW is furthermore a commodity exporter. A competitive sector supplies two types of commodities co, namely energy (ec) and non-energy (nec) materials, to foreign firms. The supply schedule follows an exogenous stochastic process

(25) $$P_{t}^{co^{*}}=\frac{1}{\varepsilon_{t}^{CO,*}}P_{t}^{*},$$

where $\varepsilon_{t}^{CO,*}$ is a commodity-specific supply shock. Preferences of RoW households include consumption with external consumption habits and endogenous labour supply. Monetary policy in the RoW follows an interest rate rule analogous to equation (20).

## 5 Econometric approach

This section describes our econometric approach. The turbulent economic dynamics in 2020-21 pose challenges for model estimation. The short-lived pandemic shocks are, however, useful when including the COVID-19 sample, as we will show below. Section 5.1 introduces the data for estimation, and Section 5.2 describes the model solution and the heteroskedastic filter. Section 5.3 summarises important estimated and calibrated parameters. We relegate a discussion of the benchmark model’s forecasting properties to Section 9 below to compare it more easily to alternative versions (robustness checks).

### 5.1 Data

We estimate the model using quarterly data from 1998q4 until 2021q4.^16^ The EA data (quarterly national accounts, fiscal aggregates, quarterly interest and exchange rates) are taken from Eurostat. Annual RoW series are based on the IMF International Financial Statistics (IFS) and World Economic Outlook (WEO) databases.^17^ We also include prices of two main commodity groups, i.e., mineral fuels and raw materials, from Eurostat Comext data as observables for energy and non-energy industrial supplies. Appendix B provides further details on the data sources and the RoW data aggregation.

### 5.2 Model solution and heteroskedastic filtering

*Linear solution*. We first construct a linear solution of the economy’s dynamic equilibrium. The model also assumes a stochastic productivity trend (the trend component of $A_{t}^{Y}$), affecting all real variables. Let Θ denote the parameters of the DSGE model. The de-trended model solution obeys the following system of state equations:

(26) $$S_{t}=\Phi_{1}\left(\Theta \right)S_{t-1}+\Phi_{\varepsilon }\left(\Theta \right)\varepsilon_{t}\;\;\varepsilon_{t}∼N\left(0,Q_{t}I\right),$$

where $\left\{\varepsilon_{t}\right\}$ collects the model shocks with time-varying co-variance matrix $Q_{t}I$, and $\Phi_{1}\left(\Theta \right)$ and $\Phi_{\varepsilon }\left(\Theta \right)$ are coefficient matrices. The system of observation equations follows

(27) $$Y_{t}^{data}=\Psi_{1}\left(\Theta \right)S_{t},$$

where $Y_{t}^{data}$ denotes the vector of observables at time t. $\Psi_{1}$ relates the model variables to the data counterparts (observation equations).

*Heteroskedastic pandemic shocks*. The state transition (26) allows for time-varying shock variances, namely

(28) $$Q_{t}=\left\{ \begin{array}{cc} Q^{COVID} & \text{for}\;t\in \{\text{2020q1:2021q4}\},\\ Q & \text{else,} \end{array}\right.$$

where Q and $Q^{COVID}$ set the shock variances. Q sets the standard deviations of the temporary COVID-specific shocks to zero before the pandemic. The approach follows Lenza and Primiceri (2022) in imposing a deterministic heteroskedasticity on the subset of COVID-related shocks for the periods 2020q1-2021q4, maintaining normally distributed errors. We estimate this specification using a heteroskedastic Kalman filter and apply a computationally efficient parallelised slice sampling algorithm (Neal, 2003) to draw parameters from their posterior distribution via Markov Chain Monte Carlo methods.^18^

Our heteroskedastic filter implies that COVID-19 unexpectedly changes the model’s probabilistic structure, i.e. new pandemic-specific shocks hit the economy starting in 2020q1. Before turning to the estimation results, it is helpful to briefly discuss alternative estimation approaches. First, one could resort to Markov-switching (MS) methods, in which case a latent process would induce time variation in the shocks. The MS setup can estimate state probabilities by filtering and smoothing methods. In addition, the MS methods allow for regime-specific parameters. The (two) states (‘pandemic’ and ‘no pandemic’) are known in our case, however. Hence, the identification process is greatly simplified because the regimes are known ex-ante, as explained in Lenza and Primiceri (2022).

A second approach would be to solve the model non-linearly and apply a non-linear filter to evaluate the likelihood. Non-linear filters can identify non-Gaussian disaster shocks. Corrado et al. (2021), e.g., use sequential Monte Carlo methods to estimate the occurrence of the (pandemic) regime. The approach has the additional advantage of accounting for model non-linearities. Yet, it is challenging to use the framework in larger models, and, similarly to the MS-DSGE approach, it does not exploit the fact that the timing of the pandemic is known.^19^ Instead, MS and non-Gaussian (fat-tail) shocks imply that agents expect disasters to happen at any time and incorporate this expectation in their decision rules. Hence, COVID-19 has not been a total surprise to agents in these frameworks. In our setting with exogenous heteroskedasticity of COVID-specific shocks, to the contrary, expectations of COVID-19 had been zero before it actually struck.

A third approach, finally, maintains the same shock structure without the modifications made in the second approach and just re-estimates the pre-COVID model with data that include the COVID-19 period so that the estimated parameters and shocks adjust to the sample extension. We discuss this option in more detail below, where we compare our benchmark model to a specification with homoskedastic shocks.

*Non-linear smoothing*. Based on the estimated parameter values from the linear model, we run a piecewise linear Kalman filter as in Giovannini et al. (2021) to identify the structural shocks until 2021q4, accounting for ELB periods. We assume that agents expect an ELB duration of four quarters in each of such periods. Imposing the ELB expected duration avoids the sensitivity of DSGE models to long ELB spells (forward guidance puzzle).^20^

### 5.3 Model parameters

*Calibration*. We calibrate a subset of model parameters to match long-run data averages or targets. All real demand components grow at the average annual growth rate of EA GDP (1.3%). The trend growth of the price level corresponds to the targeted annual inflation rate (2%). The steady-state ratios of main economic aggregates to GDP match historical averages over 1998q4–2021q4. The discount factor of 0.998 (quarterly) gives a steady-state annual real interest rate of 1%. The share of saver households is 0.67, in line with Dolls et al. (2012). The Cobb-Douglas labour share, α, equals 0.65. We calibrate the import content in aggregate demand components, $s^{M,Z}$, to Bussiere et al. (2013).^21^ The steady-state share of industrial supplies in output, $s^{IS}$, matches the average of imported commodities to GDP (0.04). The share of the energy component, $s^{ec}$, corresponds to the average share of oil in total imported commodities (0.59).

*Posterior estimates*. We estimate the remaining parameters using Bayesian full information methods applied to the linearised model. Table 1 reports estimates for a number of key parameters. Appendix B collects the estimates for the remaining parameters and processes. On the household side, relatively high consumption habits suggest a smooth consumption response to (persistent) changes in income for savers. Risk aversion and the inverse labour supply elasticity are 1.60 and 2.4, respectively. These estimates are similar to those of the literature (see, e.g., Kollmann et al., 2016). With respect to trade, the posterior mode of the import price elasticity is 1.36. We estimate a low price elasticity of EA commodity demand at around 0.32. The posterior estimates also suggest sticky prices and wages, including a large share of forward-looking price and wage setting. Key demand shocks are highly serially correlated. The estimated consumption habits in RoW are similar to the EA value. The steady-state share of liquidity-constrained firms ($\bar{sli}$) is estimated at 12%. The estimated response parameter $\zeta_{1}$ implies that changes in the firms’ gross operating surplus partly drive private investment dynamics.^22^ For the COVID-specific shocks, we set uniform priors and find relatively large shock variances.

**Table 1:** Prior and posterior distribution of key estimated model parameters.

		Prior distribution			Posterior distribution		
		Distr.	Mean	Std.	Mode	10%	90%
**Preferences**							
Consumption habit persistence	h	Beta	0.50	0.10	0.89	0.84	0.93
Risk aversion	θ	Gamma	1.50	0.20	1.60	1.35	2.01
Inverse Frisch elasticity of labour supply	$\theta^{N}$	Gamma	2.50	0.50	2.45	1.73	3.31
Import price elasticity	$\sigma^{z}$	Gamma	2.00	0.40	1.36	1.18	1.57
Oil price elasticity	$\sigma^{o}$	Gamma	0.50	0.08	0.32	0.30	0.36
**Nominal and real frictions**							
Price adjustment cost	$\gamma^{P}$	Gamma	60.00	40.00	26.21	19.69	32.60
Employment adjustment cost	$\gamma^{N}$	Gamma	60.00	40.00	0.021	0.005	0.054
Capacity utilization quadratic adj. cost	$\gamma^{u,2}$	Gamma	0.030	0.012	0.015	0.011	0.019
Investment adjustment cost	$\gamma^{I,2}$	Gamma	60.00	40.00	48.52	15.94	94.85
Nominal wage adjustment cost	$\gamma^{w}$	Gamma	15.00	3.00	16.99	10.76	22.26
Real wage rigidity	$\gamma^{wr}$	Beta	0.95	0.02	0.92	0.88	0.95
Share of forward-looking price setters	sfp	Beta	1.00	0.05	1.00	0.95	1.00
Share of forward-looking wage setters	sfw	Beta	0.50	0.20	0.90	0.73	0.96
Steady-state share of liquidity constrained firms	$\bar{sli}$	Beta	0.20	0.08	0.12	0.06	0.32
Strength of firm liquidity constraints	$\zeta_{1}$	Beta	0.10	0.04	0.09	0.03	0.14
**Fiscal policy**							
Lump sum taxes persistence	$\rho^{\tau }$	Beta	0.85	0.06	0.88	0.82	0.93
Lump sum taxes response to deficit	$\eta^{DEF}$	Beta	0.03	0.01	0.02	0.02	0.04
**Demand shock processes**							
Subjective discount factor - AR(1) coeff.	$\rho^{C}$	Beta	0.50	0.20	0.74	0.51	0.87
Subjective discount factor - std. (%)	$\varepsilon^{C}$	Gamma	0.010	0.004	0.010	0.004	0.012
Investment risk prem. - AR(1) coeff.	$\rho^{S}$	Beta	0.50	0.20	0.90	0.88	0.95
Investment risk prem. - std. (%)	$\varepsilon^{S}$	Gamma	0.008	0.004	0.010	0.003	0.013
**RoW region**							
Habit persistence	$h^{*}$	Beta	0.70	0.10	0.95	0.92	0.96
Risk aversion	$\theta^{*}$	Beta	1.50	0.20	1.37	1.05	1.41
Elasticity mineral fuels and raw materials	$\sigma^{C^{*}}$	Gamma	2.00	0.40	1.11	1.04	1.19
Price adjustment cost	$\gamma^{P^{*}}$	Gamma	60.00	40.00	14.68	10.11	33.28
**COVID-related shocks (standard deviation)**							
Forced saving shock	$\varepsilon^{tC}$	Uniform	0.05	0.03	0.04	0.03	0.07
Labour hoarding shock	$\varepsilon^{tN}$	Uniform	0.005	0.003	0.0100	0.0096	0.0100

## 6 Propagation of key shocks

This section presents impulse response functions (IRFs) for key shocks to better understand the dynamics of the model given the parameter estimates. In particular, Section 6.1 highlights the transmission of the lockdown (‘forced savings’) shock compared to the standard savings (time preference) shock. Furthermore, Section 6.2 discusses the transmission of investment risk, labour hoarding, wage markup, and trade shocks, which are also prominent in the shock decompositions presented in Section 7. In light of our focus on the COVID-19 period (2020-21), we consider a scenario in which the ELB on the short-term nominal interest rate in the EA binds for one year.

### 6.1 COVID-specific shocks

**Fig. 2:**
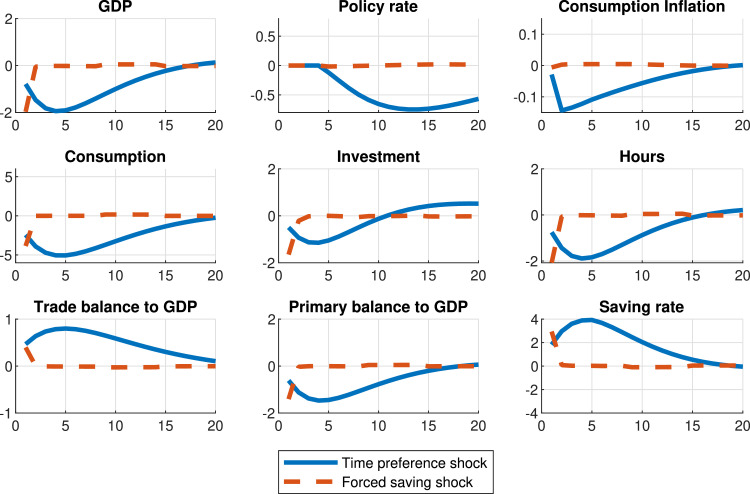
Dynamics of transitory (‘forced’) and persistent saving shocks. *Notes:* All variables are displayed in percent deviations from steady state, except for the trade balance relative to GDP, the government's primary balance relative to GDP, the policy rate (annualised), quarterly consumption inflation, and the household saving rate, which are expressed in percentage-point deviations from steady state instead. Periods correspond to quarters. The size of the ‘forced saving’ shock corresponds to the identified shock in 2020q1. The persistent saving shock is scaled to generate a similar GDP response (matching the trough). The IRFs are state-dependent in light of the occasionally binding ELB and initialised at the state of 2019q4. To recover the state-dependent IRFs, we subtract the effects of the initial conditions - obtained by running a simulation using only the initial conditions without any shock - from the shocked path. The saving rate refers to private savings, i.e. net household income minus private consumption.

Figure 2 compares the transmission of the non-persistent ‘forced savings’ shock to the standard savings (time preference) shock in the model. The non-persistent ‘forced savings’ shock lowers consumption for one period. Lower consumption demand, together with firm financing constraints and the absence of monetary stimulus, also reduces investment demand. Real GDP declines temporarily, which triggers a temporary decline in actual hours worked of similar size. The trade balance increases in the short run due to lower domestic and import demand, and the government’s primary balance deteriorates in response to lower tax revenue and growing benefit payments. The economy’s initial response to the standard saving shock is qualitatively similar. However, the standard savings shock is subject to habit persistence, leading to a hump-shaped pattern in domestic demand. In addition, the standard savings shock itself is persistent and is still active when the economy exits the ELB environment after four quarters. The central bank then reacts with a policy rate reduction in the medium term relative to the no-shock baseline, which stabilises private investment and leads to domestic currency depreciation, which leads to an increase in the trade balance.^23^

**Fig. 3:**
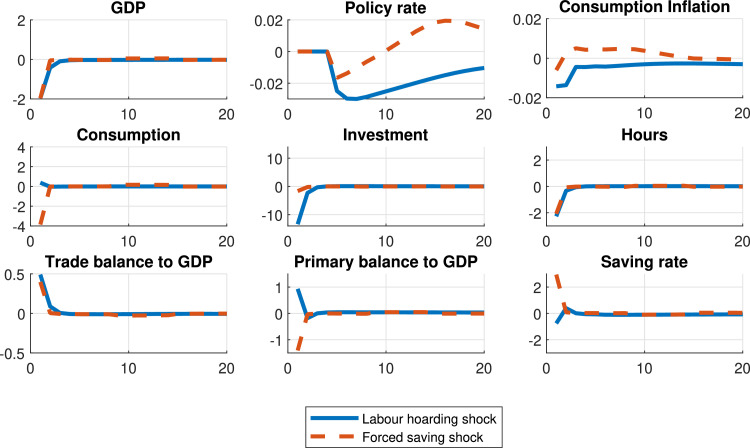
Dynamics of transitory ‘forced saving’ and labour hoarding shocks. *Notes:* All variables are displayed in percent deviations from steady state, except for the trade balance relative to GDP, the government's primary balance relative to GDP, the policy rate (annualised), quarterly consumption inflation, and the household saving rate, which are expressed in percentage-point deviations from steady state instead. Periods correspond to quarters. The labour hoarding shock is scaled to generate a GDP response that is similar to the savings shock. The IRFs are state-dependent in light of the occasionally binding ELB and initialised at the state of 2019q4. To recover the state-dependent IRFs, we subtract the effects of the initial conditions - obtained by running a simulation using only the initial conditions without any shock - from the shocked path. The saving rate refers to private savings, i.e. net household income minus private consumption.

Figure 3 compares non-persistent labour hoarding as the second COVID-specific shock to the ‘forced savings’ shock from Fig. 2. Labour hoarding is a non-persistent shock to labour demand so that effective hours worked decline. Investment falls because the marginal return to capital declines as the capital-labour ratio increases and because of liquidity-constrained investment that reacts to the decline in turnover. The savings rate declines temporarily; households maintain real consumption levels while overall income (GDP) declines. Inflation declines temporarily in line with lower output and lower wage pressure.

In sum, IRFs for the COVID-specific shocks show qualitative patterns that can explain salient features of the pandemic period (Fig. 1). ‘Forced savings’ sharply reduce consumption demand, followed by a swift recovery. Private investment and economic activity decline, and the government fiscal balance deteriorates (increase in the government debt-to-GDP ratio). Labour hoarding fills the gap between declining hours worked and rather stable employment in persons during the pandemic. Neither transitory savings, nor labour hoarding imply a (significant) change in inflation. Both shocks increase the EA trade balance, contrary to what is observed in the data. Hence, fitting the information from our rich data set requires additional shocks, including COVID-specific ones in the RoW block. Section 6.2 sketches other key drivers of EA activity during COVID-19.

### 6.2 Other main shocks

**Fig. 4:**
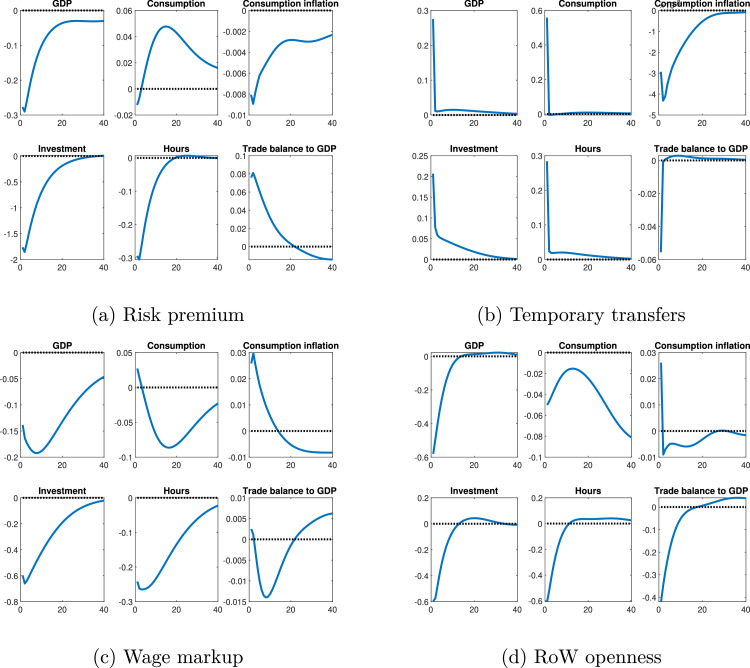
IRFs of key shocks. *Notes:* All variables are displayed in percent deviations from steady state, except for the trade balance to GDP and quarterly consumption inflation which are expressed in percentage-point deviation. Periods on the x-axis are quarters. The IRFs are initialised at the state of 2019q4. Shock sizes are normalised to one standard deviation.

Figure 4 summarises IRFs for four other shocks that the estimation identifies as relevant drivers of macroeconomic dynamics during COVID-19, as will be shown in Section 7. The risk premium shock in Panel (a) is a temporary increase in investment risk, with an estimated degree of persistence. Higher investment risk (financing costs) reduces investment demand. Real GDP and hours worked decline. The real GDP reduction is very persistent as less investment and capital lower the production potential in the medium and longer term. Net exports temporarily increase as a result of lower import demand and real effective depreciation. Consumption, to the contrary, increases moderately, given that saver households lower investment and increase consumption instead.

Panel (b) plots IRFs for a temporary increase in government transfers. Private consumption increases, driven by stronger spending by hand-to-mouth households. Net exports decline with growing import demand. Investment increases with consumption and economic activity due to additional investment by financially-constrained firms.

The wage markup shock in Panel (c) raises wages and, consequently, lowers hours worked and real GDP. Investment also declines because of the firms’ financing constraints and because lower employment reduces the marginal return to capital. Consumption increases initially with the increase in wages but declines thereafter in line with falling total income.

The RoW home bias shock in Panel (d), finally, reduces the degree of RoW trade openness, which implies lower EA export demand. The trade balance, economic activity, and employment in the EA decline. Persistently lower exports reduce investment demand as the firms’ financing constraint tightens. Consumption declines modestly, driven by the negative impact of lower employment on the disposable income of liquidity-constrained (‘hand-to-mouth’) households.

## 7 Drivers of the COVID-19 recession

This section focuses on the main drivers of EA economic fluctuations, which we infer from shock decompositions (SDs) of real GDP growth and inflation at a quarterly frequency. The SDs attribute the dynamics of endogenous variables to the various estimated exogenous shocks. To play an important role in SDs, a shock must capture important data patterns not only for the particular variable of interest but also for the entire set of observables. In particular, our rich two-region model informs about the quantitative importance of domestic demand and supply factors as well as global economic conditions.

Section 7.1 presents results from the augmented (‘benchmark’) model version described in Section 4, which includes the COVID-specific (‘forced savings’, labour hoarding) shocks and liquidity-constrained firms. Section 7.2 contrasts the findings to SDs of GDP growth from the same model without COVID-specific shocks.

### 7.1 Model with COVID-specific shocks

**Fig. 5:**
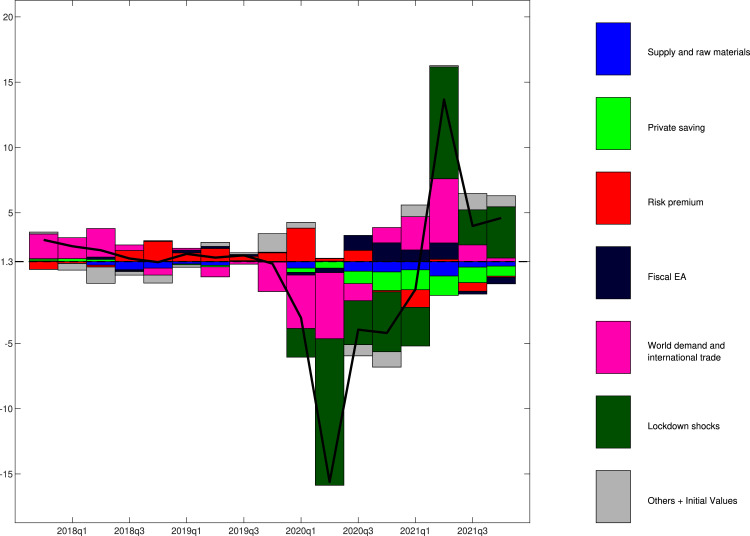
EA real GDP growth (per cent, year-on-year). *Notes:* The figure shows the shock decomposition of year-on-year real GDP growth for 2017q4–2021q1. All structural shocks together recover the observed time series of GDP growth (continuous black line). We have grouped the estimated shocks into seven broad categories: (1) supply shocks and raw materials, including TFP, price and wage markup shocks (blue); (2) persistent private saving shocks (light green); (3) investment risk premium shocks (red); (4) discretionary fiscal policy shocks, which capture deviations from estimated fiscal policy rules (black); (5) shocks to world demand and international trade, which include foreign demand and supply shocks as well as deviations of trade volumes and prices from the estimated export and import demand and pricing equations (pink); (6) lockdown shocks since 2020 (‘forced saving’ shock, labour hoarding shock) (dark green); (7) other remaining factors (grey). (For interpretation of the references to colour in this figure legend, the reader is referred to the web version of this article.)

*GDP growth*. Figure 5 provides a quantitative assessment of the drivers of year-on-year real GDP growth during the period 2017q4–2021q1. The COVID-specific shocks, labelled ‘lockdown shocks’ in the figures, have been the dominant driver of the 2020 recession, with a strong contraction in 2020q2, an easing in 2020q3 (improvement compared to 2020q2), and a renewed deterioration in 2020q4 that reverts in 2021. Among these shocks, ‘forced savings’ dominate the picture, whereas the labour hoarding shock plays only a minor role in GDP dynamics. A persistent increase in household savings (‘private savings’ in Fig. 5) has added to the massive decline in private consumption as the most important component of the 2020 downturn. The role of the persistent savings shock increased in the second half of 2020 and continued to weigh negatively on growth in 2021, contrary to the sign flip of the ‘forced savings’ contribution. The persistent saving shock may reflect precautionary savings due to elevated uncertainty about the pandemic dynamics and its economic impact, and the fact that restrictions have become more entrenched with the duration of COVID-19. The impact on global demand and trade has been a third important driver of the 2020 contraction of economic activity, with falling export demand and some offsetting from stronger home bias on the import side. The recovery of global demand has turned the group of shocks into a growth supporting factor starting in 2020q4 but fading towards the end of the sample. Investment demand (‘risk premium’) shocks play only a minor role in 2020-21, illustrating that the extension by firm liquidity constraints fits the investment dynamics well. Standard supply factors as a group are equally of little importance. Discretionary fiscal stimulus supported GDP growth in the second half of 2020 and early 2021, and it complemented the working of automatic stabilisers that are present in the model. The contribution of fiscal shocks remains quantitatively moderate, however, in line with the nature of the stimulus, i.e. mainly transfers in an environment in which the transfer multiplier is modest (only one-third of hand-to-mouth consumers).

**Fig. 6:**
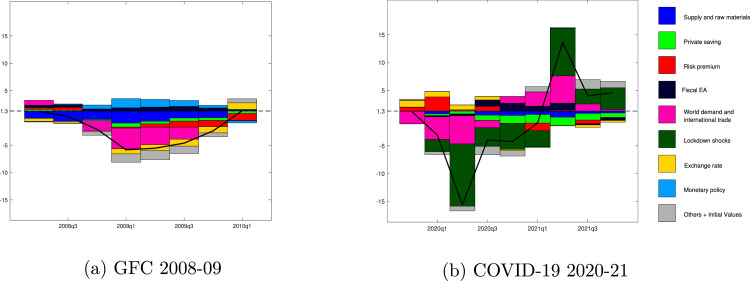
EA real GDP growth in GFC and COVID-19 recessions (per cent, year-on-year). *Notes:* The panels show shock decompositions of quarterly real GDP growth in the EA during the Global Financial Crisis (GFC) and the COVID-19 crisis recession. All structural shocks together recover the observed time series of GDP growth (continuous black line). We have grouped the estimated shocks into nine broad categories: (1) supply shocks and raw materials, including TFP, price and wage markup shocks (blue); (2) private saving shocks (light green); (3) investment risk premium shocks (red); (4) discretionary fiscal policy shocks, which capture deviations from estimated fiscal policy rules (black); (5) shocks to world demand and international trade, which contain foreign demand and supply shocks as well as deviations of trade volumes and prices from the estimated export and import demand and pricing equations (pink); (6) lockdown shocks since 2020 (‘forced saving’ and temporary labour hoarding shocks) (dark green); (7) exchange rate shocks (yellow); (8) monetary policy (light blue); (9) any remaining factors (grey). (For interpretation of the references to colour in this figure legend, the reader is referred to the web version of this article.)

To compare the COVID-19 recession to the GFC of 2008-09, Fig. 6 zooms into both episodes with SDs of year-on-year (y-o-y) real GDP growth. An obvious difference is that the slowdown of real GDP growth during the GFC has been more persistent than the more volatile growth performance during COVID-19. The two episodes also differ with respect to the main drivers. Lower world demand and international trade, lower investment demand and the appreciation pressure on the euro explain a large part of the GFC recession, whereas discretionary monetary easing and fiscal expansion mitigated its depth. The SD for the COVID-19 recession, to the contrary, assigns a much larger role to domestic consumption, notably the transitory (‘forced’) savings shock. The other important driver in 2020-21 is the decline and subsequent recovery of world demand and international trade. Discretionary fiscal policy contributed positively to GDP growth.

**Fig. 7:**
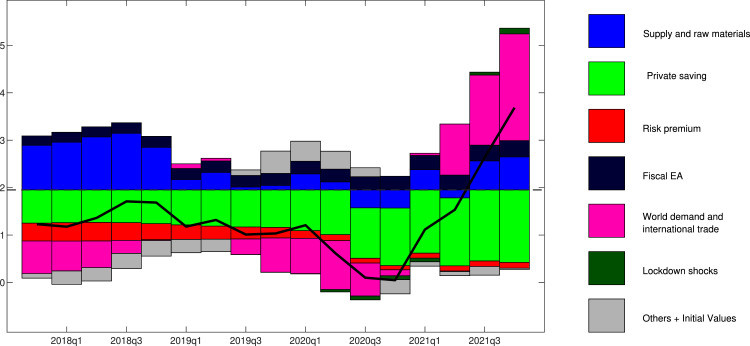
EA inflation (per cent, year-on-year). *Notes:* Inflation rates are year-on-year, i.e. they measure consumer price deflator growth relative to the same period of the previous year. Units on the y-axis are % (1=1%). The solid black line represents the data. Bars below (above) the dashed line (trend inflation) indicate negative (positive) contributions to consumer price inflation. All structural shocks together recover the observed time series of GDP growth (continuous black line). Estimated shocks are grouped into seven broad categories: (1) supply shocks and raw materials, including TFP, price and wage markup shocks (blue); (2) persistent private saving shocks (light green); (3) investment risk premium shocks (red); (4) discretionary fiscal policy shocks, which capture deviations from estimated fiscal policy rules (black); (5) shocks to world demand and international trade, which contain foreign demand and supply shocks as well as deviations of trade volumes and prices from the estimated export and import demand and pricing equations (pink); (6) lockdown shocks since 2020 (transitory ‘forced’ saving and labour hoarding shocks) (dark green); (7) other remaining factors (grey). (For interpretation of the references to colour in this figure legend, the reader is referred to the web version of this article.)

*Inflation*. The drivers in the SD for EA consumer price inflation (Fig. 7) differ from those in the SD for real GDP growth. Transitory lockdown shocks have little impact on current inflation, despite strong negative output effects, because they do not alter inflation expectations. Figs. 2 and 3 in Section 6.1 have illustrated this property of the transitory shocks. Persistent adverse saving shocks (‘private saving’), in contrast, play a stronger role (in relative terms) than in the GDP decomposition. The reason is that persistent negative demand shocks lower inflation expectations, which translates into lower actual inflation in an economy with sticky prices and forward-looking pricing behaviour. The contribution by persistent private savings shocks reflects the pre-COVID trend of persistently high savings. It does not explain the increase in inflation in 2021, however. Expansionary fiscal policy has been a positive driver of inflation in 2021 but remains modest compared to international and supply-side contributions, as discussed next.

The international environment is central to EA inflation. Shocks to world demand and international trade, which also display some persistence, weigh negatively on inflation in 2020 and positively in 2021, where they are the main force behind the rise in EA consumer price inflation. This group of shocks includes the recovery in the RoW (including the US). In the last quarter of 2021, the model also identifies a large export price shock in the RoW, possibly reflecting supply-chain bottlenecks. Within the group of ‘supply and raw materials’ shocks, commodity prices account for most of the acceleration of consumer price inflation in 2021. Adverse productivity shocks in the EA also increase consumer prices while lowering economic activity. Thus, while the initial inflation response has been modest compared to the recession’s severity, the contribution of persistent shocks (persistent savings, foreign shocks, commodity shocks) increases over time. Overall, the impact of international factors and commodity prices has been exceptionally strong in the second half of 2021, suggesting that a large part of the increases in EA inflation in 2021 is imported.

### 7.2 Model without COVID-specific shocks

What is the value added of the COVID-specific model extensions compared to the pre-COVID set-up (‘standard GM model’) in Albonico et al. (2019)? How do the COVID-specific shocks affect the economic interpretation of the COVID-19 recession? This subsection provides answers by comparing SDs for EA real GDP growth (quarter-on-quarter) from three variants of the model, i.e. (a) the model version without the COVID-related shocks and with parameters estimated on data until 2019q4, (b) the model without the COVID-related shocks and with parameters estimated on data until 2021q4, and (c) the benchmark version of this paper with the COVID-specific extensions and estimation with the full sample.

**Fig. 8:**
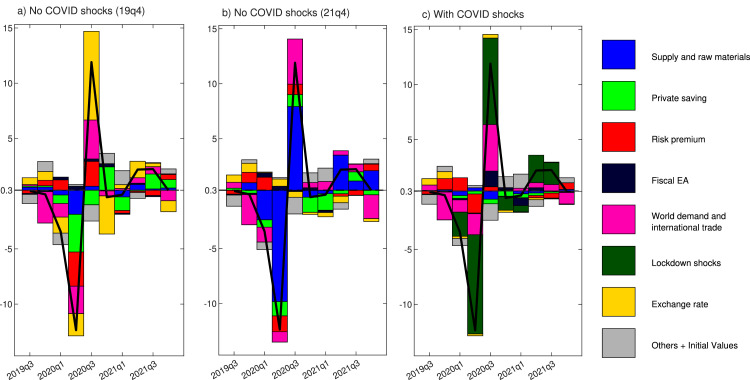
EA real GDP growth across model variants (per cent, quarter-on-quarter). *Notes:* The first two panels show shock decomposition of real GDP growth in the EA (quarter-on-quarter) in a models without COVID-shocks estimated until 2019q4 (a) and 2021q4 (b), respectively. Panel (c) shows the model with COVID-specific shocks and extensions. See Fig. 6 for additional details on the shock grouping.

Figure 8 compares the results from this three model variants. The plots look relatively similar with respect to the role of foreign and investment demand shocks. There are important differences w.r.t. consumption shocks and exchange rate shocks, however. The augmented model (c) attributes a large part of the drop in consumption in 2020q1-2 to the ‘forced savings’ shock. As the ‘forced savings’ shock is non-persistent, it can fit the large contraction and partial recovery of private consumption in 2020q3. Similarly, in this model, one can expect consumption to recover quickly in a forecasting context (‘pent-up demand’).

Without the COVID-specific shocks, model (a) explains the drop in consumption by the standard (‘voluntary’) saving shocks, i.e. a decline in the rate of time preference. This standard saving shock is persistent, however, and consumption responds with strong habit persistence. A persistent saving shock is difficult to reconcile with a quick recovery in consumption demand, as illustrated in Fig. 2 above. Instead, the standard pre-COVID model in Panel (a) implies large exchange rate and, to a lesser extent, wage markup shocks in 2020q2. The exchange rate shocks are needed to reconcile observed exchange rates with the expected future path of (real) interest rates. In particular, the persistent negative demand shocks in 2020q2 would suggest a path of lower interest rates that needs then to be combined with a currency appreciation shock to match the observed euro exchange rate. Similarly, the recovery in 2020q3 fit with persistent shocks would suggest a persistent path of higher domestic interest rates. The euro exchange rate is then matched with a depreciation shock. The positive wage markup shock in 2020q2 is needed to reconcile the decline of hours worked with the stability of the compensation of employees in the absence of labour hoarding shocks. The wage markup shock has the impact of a negative supply shock and lowers GDP growth, as shown in Fig. 4 above.

When re-estimated until 2021q4, the model without COVID-specific shocks provides a markedly different growth decomposition. Panel (b) assigns a dominant role to domestic supply-side factors to explain growth fluctuations in 2020. In the absence of temporary labour hoarding, a large positive wage markup shock is required in 2020q2 to reconcile the drop in actual hours worked with a comparatively stable compensation of workers, which then lowers GDP growth in the model (Fig. 4). Estimation over the entire sample lowers the persistence of the standard (‘voluntary’) savings shock, but the latter remains a minor factor in the decomposition in Panel (b) of Fig. 8.

### 7.3 Forecast revisions

Another way of looking at the macroeconomic implications of COVID-19 is the analysis of forecast errors, as suggested by Kollmann (2021). COVID-19 has been a very large exogenous shock and arguably the primary driver of discrepancies between the 2019 EA forecast for 2020-21 and the realisations.

**Fig. 9:**
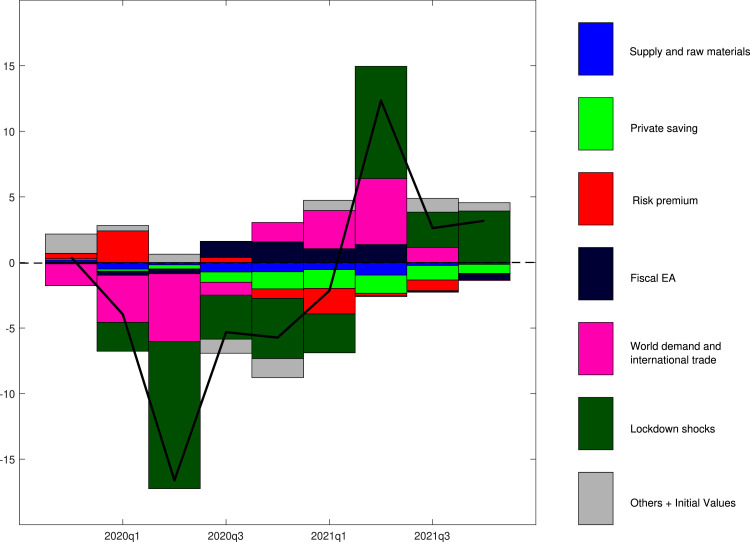
Forecast error for EA real GDP growth (per cent, year-on-year). *Notes:* The chart shows the forecast error as the difference between EA real GDP growth (year-on-year) and the forecast for EA real GDP growth (year-on-year) from Autumn 2019. Units on the y-axis are percentage points (1=1pp forecast error). The solid black line shows the forecast error. Bars below (above) the zero line indicate negative (positive) contributions to the forecast error. The grouping of shocks corresponds to Fig. 5.

Figure 9 provides a shock decomposition of the forecast error for real GDP growth. More precisely, we decompose the difference between EA real GDP growth (year-on-year) in 2020q1-2021q4 as reported in Spring 2022 and the corresponding European Commission Autumn 2019 forecast (European Commission, 2019).^24^

Figure 9 gives a picture that is similar to the message from Fig. 5. COVID-specific shocks, notably ‘forced savings’, are the main driver of the COVID-related forecast error, together with an upward revision of persistent savings (i.e. a persistent downward revision of private consumption growth), and negative surprises for world demand and trade in 2020, followed by a recovery of external demand in 2021. Discretionary fiscal policy was more expansionary during 2020-21 than expected in 2019 due to the fiscal response to the pandemic, which explains the positive contribution of fiscal shocks to growth revisions in the second half of 2020 and early 2021.

## 8 Evidence for COVID-specific shocks

The transitory ‘forced savings’ and labour hoarding shocks play a major role in explaining EA GDP growth and employment during the COVID-19 crisis. Section 8.1 shows that the identified shock processes broadly match indicators of COVID-related restrictions that are not part of our estimation data set. As an additional test, Section 8.2 allows for the presence of the ‘forced savings’ shock over the whole sample period and assesses the ability of the estimation to identify this shock without the restriction of pre-COVID zero variance in our benchmark setting.

### 8.1 Shock profiles and external indicators

**Fig. 10:**
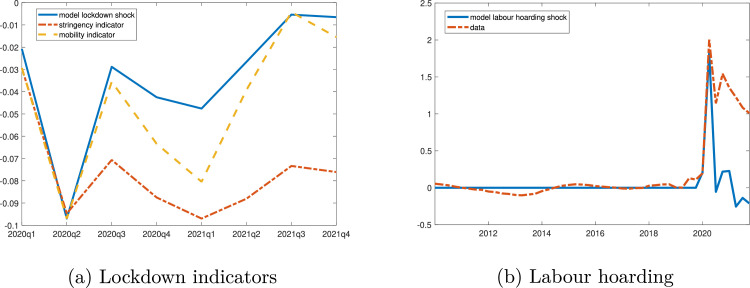
Model shocks and indicators. *Notes:* In Panel a) the model shock (blue solid line) corresponds to the estimated ‘forced savings’ shock as described in Eq. (16). The data are time series of the Oxford stringency index (Hale et al., 2020) (red dot-dashed line) and Google’s mobility indicator (yellow dashed line), aggregated across EA countries (both with maximum absolute scaling). In Panel b) the data correspond to demeaned subsidies (on labour) received by the employer as a share to GDP (red dot-dashed line). The model shock corresponds to the estimated ‘labour hoarding shock’ as shown in Eq. (8). (For interpretation of the references to colour in this figure legend, the reader is referred to the web version of this article.)

The decomposition of macroeconomic dynamics in estimated DSGE models depends on the estimated shocks. The latter often remain rather abstract and difficult to interpret or to compare to real-world equivalents.^25^ In Fig. 10, we compare the smoothed estimates of the COVID-specific shocks to indicators that are not part of the data used for model estimation. In particular, Panel (a) plots the transitory ‘forced savings’ shock, which is the most important individual driver of the quarterly profile of economic activity in 2020-21, together with empirical measures of lockdown stringency and mobility restrictions. The shapes are similar, with a particularly close co-movement between the estimated shock and the mobility indicator at least in 2020. The co-movement suggests that ‘forced savings’ in the model reflect contact restrictions and supply constraints. Consumption recovers more strongly than the restriction and mobility indices during the second half of 2020, possibly reflecting increasing reliance on online retail. The two indicators and the estimated shock also move in parallel during the renewed worsening of the epidemiological situation in late 2020 and the subsequent recovery in 2021.

Panel (b) of Fig. 10 compares short-time work as identified via government wage subsidies in the data together with the model’s labour hoarding shock, which has been introduced to account for the discrepancy between the decline in actual hours worked and comparatively stable employment in persons and compensation of workers in the EA during COVID-19.

### 8.2 Forced savings in the full sample

**Fig. 11:**
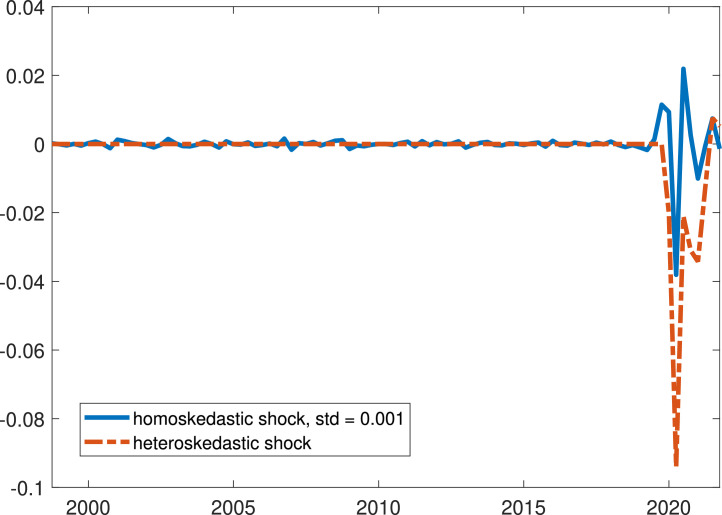
Smoothed estimates of ‘forced saving’ innovations. *Notes:* The red dashed line refers to the benchmark model (no ‘forced saving’ shock prior to 2020) and the blue solid one to the model with estimated homoskedastic ‘forced saving’ shock. (For interpretation of the references to colour in this figure legend, the reader is referred to the web version of this article.)

**Fig. 12:**
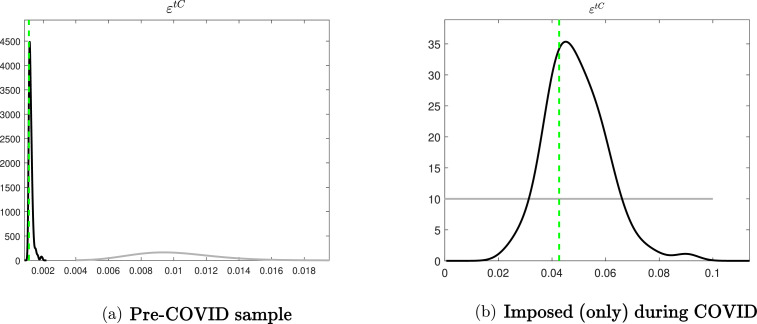
Prior and posterior of the forced saving shocks (shock standard deviation). *Notes:* This figure plots the prior (grey) and posterior (black) densities of the forced saving shock estimated (a) before COVID and (b) imposed (only) during COVID. The y-axes show the corresponding densities. The green lines indicate the posterior mode. The estimation includes five quarters of pre-sample starting in 1998q4.

In the benchmark model results in Section 7, the transitory ‘forced savings’ shock enters with a large standard deviation only in 2020q1. Fig. 11 plots the smoothed innovations starting in 2000q1. The shock profile for the benchmark model corresponds to the red line. To test the appropriateness of imposing heteroskedasticity in the shock, with zero standard deviation until 2019q4, we estimate a model variant that includes the transitory ‘forced saving’ shock on data until 2019q4. The estimated standard deviation of the shock during 2000q1–2019q4 is 25 times smaller than in the benchmark model, where the shock is restricted to 2020q1-2021q4, as shown in Panel (a) of Fig. 12.

The blue line in Fig. 11 displays the profile of the estimated ‘forced saving’ shock when we extend the sample to retrieve the 2020q1-2021q4 shocks, based on the parameter estimates from the 2000q1-2019q4 sample, with the smaller estimated shock variance. The profile of the blue line suggests that transitory ‘forced savings’ shocks are irrelevant before 2020 compared to the COVID-19 recession. The estimated profile resembles a white noise shock without any pre-2020 spike comparable in magnitude to the 2020-21 innovations. In particular, the ‘forced savings’ shock shows no comparable spike around the GFC or the EA sovereign debt crisis. We conclude that the transitory ‘forced savings’ shock in the model is particular to the COVID-19 crisis, supporting our heteroskedastic specification.

## 9 Model fit and alternative shock specifications

The central ingredient of our benchmark model is a simple specification of ‘forced savings’ shocks. Section 9.1 discusses econometric results supporting this feature. The ‘forced savings’ shock captures the unexpected extreme contraction and sharp consumption rebound in the first COVID-19 wave (2020q2-3). Nonetheless, Sections 9.2 and 9.3 present alternative shock specifications that can help to further improve the fit of consumption and output during the pandemic. In particular, we consider a temporary time preference shock with a moving average structure and a more complex wave-specific shock structure, and we compare their forecasting performance to the benchmark model. Appendix C.2 reports additional measures of fit and discusses parameter stability.

### 9.1 Model fit

*Marginal likelihood*. Table 2 illustrates that the transitory COVID-specific shocks improve the econometric fit of the model substantially, as measured by marginal likelihood statistics (Appendix C reports further measures). The first row of the table reports the marginal likelihood of different model specifications for the full sample, i.e. including the COVID-19 period. The second row reports marginal likelihood computations using only pre-COVID data (irrespective of the estimation sample). The log marginal likelihood of our benchmark model with ‘forced savings’ is the highest (9024.1) and more than 250 log points higher than the model without these shocks. If, in addition, the model estimation (without COVID-specific shocks) only uses data until 2019q4, the likelihood computed with data including the COVID-19 period falls dramatically.^26^ Interestingly, even when computing the marginal likelihood only using pre-COVID data, the model with ‘forced savings’ is only 6 log points below the unadjusted baseline GM model (i.e. without COVID-specific shocks) fitted to this period. According to the marginal likelihood measure, the model with ‘forced savings’ also (marginally) outperforms the more complex COVID-shock specifications outlined below.

**Table 2:** Marginal likelihood across model specifications.

	base (est. 19q4)	base (est. 21q4)	FS	DF	FS+DF
2000Q1 : 2021Q4	996.2	8769.1	9024.1	8969.7	8982.8
2000Q1 : 2019Q4	8551.3	8303.9	8545.1	8505.1	8515.0

*Notes:* The marginal likelihood is computed using the modified harmonic mean method (Geweke, 1999). The first two columns report results of the baseline GM model without COVID-specific shocks, estimated using data until 2019q4 and 2021q4, respectively. Columns ‘FS’ and ‘DF’ report results from our benchmark model with ‘forced savings’ and the MA(1)-discount factor shock specification outlined in Section 9.2. The last column shows results from the mixture model (‘FS + DF’).

**Fig. 13:**
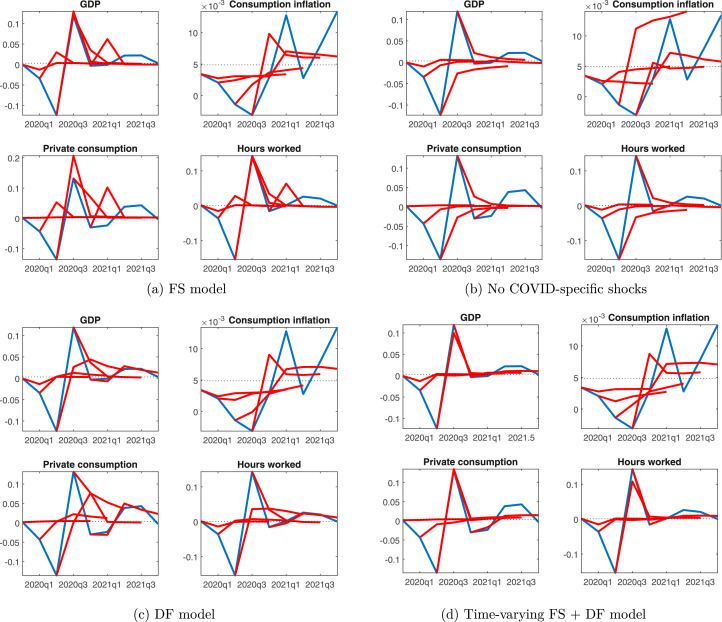
Recursive forecasting performance. *Notes:* The four panels show recursive forecasts for the quarterly growth rates of GDP, consumption inflation, private consumption, and hours worked. 0.01 corresponds to 1 percentage point. Panels (a) and (b) report results from our benchmark model with ‘forced savings’ and the model without COVID shocks (estimated until 2021q4). Panel (c) shows results from a version with temporary MA(1) discount factor shock, but without transitory ‘forced savings’ shock. Panel (d) considers the rich specification allowing for wave-specific discount factor shocks *and* transitory ‘forced savings’ shocks.

*Recursive forecasts*. Fig. 13 shows that the ‘forced savings’ model depicted in Panel (a) successfully captures the economic dynamics observed during the first COVID-19 wave, i.e. an unexpected deep contraction in 2020q2, followed by a fast recovery in 2020q3. This pattern is noticeable in GDP and the growth rates of hours worked and private consumption. By contrast, the model without COVID-specific shocks, estimated using data until 2021q4 and shown in Panel (b), fails to predict the fast recovery of these variables. Note, however, that the forecast error of the forced-savings model (Panel a) remains relatively large outside 2020q2-3. The model predicts an immediate rebound following the initial COVID-19 outbreak in March 2020. As a result, the model requires a larger shock in 2020q2, which over-predicts the consumption rebound in 2020q3. Moreover, the model does not fully capture the persistent adverse effects in 2021.

### 9.2 Alternative model I: Temporary discount factor shocks

The first alternative model adds another time preference shock, $\varepsilon_{t}^{TB}$, to our standard time preference process $\varepsilon_{t}^{C}$. The specification could, in principle, address the missing persistence of the simple ‘forced saving’ shock. In particular, unlike the transitory ‘forced saving’ shock, the discount factor shocks ($\varepsilon_{t}^{TB}$ and $\varepsilon_{t}^{C}$) are subject to habit persistence. Formally, we modify the disturbance of the discount factor $\xi_{t}$ in the utility function (Eq. (16)) as follows:

$$\log\left(\xi_{t+1}/\xi_{t}\right)=\left(\varepsilon_{t}^{C}+\varepsilon_{t}^{TB}\right),\;\varepsilon_{\tau }^{TB}∼MA\left(1\right),$$

where, in line with Chen et al. (2020), $\varepsilon_{t}^{TB}$ follows a 1st-order moving average. The MA(1) specification implies a carryover of present innovations into the near future, i.e. current innovations affect the shock term one quarter ahead, contrary to the longer-lasting AR(1) processes embedded in $\varepsilon_{t}^{C}$. We then replace our ‘forced saving’ shocks and re-estimate the model over the full dataset. As before, the heteroskedastic filter constrains the novel shock to the COVID-19 period.

Panel (c) in Fig. 13 shows that this specification implies excessively persistent growth surprises in 2000. The projections suggest that the transitory ‘forced saving’ shock better replicates the V-shape of quarterly real GDP growth during the first wave of the pandemic. By contrast, the temporary discount factor shocks capture the persistent effects outside this period. The MA(1) specification introduces persistence in COVID-specific shocks, capturing persistence in the second half of 2020 and early 2021. The implied profile of COVID-specific shocks differs strongly from the mobility and stringency indicators shown in Fig. 10, however.

### 9.3 Alternative model II: A wave-specific mixture model

The discussion in Sections 9.1 and 9.2 has emphasised two observations, i.e. the extreme V-shape contraction and rebound in 2020q2-3, and the rather persistent savings effects in subsequent quarters of the pandemic. The transitory ‘forced savings’ shock better explains the first wave. The MA(1) time preference shock gains prominence during the subsequent waves in late 2020 and 2021. These periods have been associated with more entrenched restrictions on economic activity. The prolongation of restrictions could be expected weeks or months in advance, given the epidemiological dynamics.

In light of the merits of each set-up, the final specification combines the two models. Transitory ‘forced savings’ shocks are assumed to occur in 2020q2, while the persistent shocks capture the subsequent entrenched pandemic effects.^27^ As shown in Panel (d) in Fig. 13, this mixture model is the most successful one in replicating key patterns of consumption and output growth throughout the pandemic.

## 10 Conclusion

This paper has estimated a two-region (EA, RoW) DSGE model to analyse the COVID-19 recession in the EA. We have augmented the European Commission’s Global Multi-country model (Albonico, Calais, Cardani, Croitorov, Ferroni, Giovannini, Hohberger, Pataracchia, Pericoli, Pfeiffer, Raciborski, Ratto, Roeger, Vogel, 2019, Giovannini, Hohberger, Kollmann, Ratto, Roeger, Vogel, 2019) with shocks and channels that were particularly relevant during the COVID-19 pandemic. These elements include transitory ‘forced savings’ and labour hoarding shocks, which capture the impact of temporary restrictions on demand or supply and the gap between employment and hours worked (short-time work), respectively, and firm liquidity constraints that link investment demand to firm profits.

Hence, we have chosen a parsimonious way to adapt workhorse DSGE models of policy institutions to salient features of the COVID-19 recession. The model extensions allow to estimate our model on data including the pandemic period, while preserving plausible model dynamics prior to and during the 2020-21 period. Econometric model validation, the assessment of model fit, and the comparison with alternative specifications support our pragmatic approach.

Shock decompositions have highlighted the importance of COVID-specific shocks, in particular ‘forced savings’, as the main driver of real GDP growth fluctuations at quarterly frequency in 2020-21. ‘Forced savings’ are transitory, contrary to the persistent standard savings shock, which appeared to become more important in the second half of 2020, and which can be linked to precautionary motives and entrenched restrictions. The estimated profile of the ‘forced saving’ shock co-moves with empirical indicators of the stringency of restrictions and mobility. The global nature of the COVID-19 pandemic has contributed to the recession through the decline in world demand and trade, followed by a recovery. Discretionary fiscal policy had a stabilising impact on EA GDP in 2020-21. Fiscal multipliers are modest in light of the predominance of transfers to households, however. Instead of fostering aggregate demand by government spending, stabilisation policy has focused on income support and maintaining the productive infrastructure of the economy (short-term work, firm support) in order to contain negative spillover to non-confined sectors, limit scars and facilitate the rebound.

## Figures and Tables

**Table B.1:** List of observables.

Euro Area	
i	Nominal short term interest rate
log(actr)	Log of active rate population
$\log(\frac{B^{G}}{Y})$	Log of nominal gov. bonds share
$\log(\frac{C^{G}}{Y})$	Log of nominal gov. consumption share
$\log(\frac{C}{Y})$	Log of nominal consumption share
log(e)	Log effective nominal exchange rate
$\log(\frac{i^{G}}{Y})$	Log of nominal gov. interest payments share
$\log(\frac{I^{g}}{Y})$	Log of nominal gov. investment share
$\log(\frac{I}{Y})$	Log of nominal investment share
$\log(\frac{M}{Y})$	Log of nominal total import share
log(N)	Log of hours
$\log(\frac{P^{c,vat}}{P})$	Log of consumption price final to observed GDP price
$\log(\frac{P^{g}}{P})$	Log of gov. observed price to observed GDP price
$\log(\frac{P^{IG}}{P})$	Log of govt. investment price to observed GDP price
$\log(\frac{P^{I}}{P})$	Log of observed total investment price to observed GDP price
$\log(\frac{P^{M}}{P})$	Log of import price to observed GDP price
log(Pop)	Log of population
$\log(\frac{P^{X}}{P})$	Log of export price to GDP price
log(P)	Log of observed GDP price
$\log(\bar{tfp})$	Log of TFP trend
$\log(\frac{T}{Y})$	Log of nominal gov transfers share
$\log(\frac{W}{Y})$	Nominal wage share
$\log(\frac{X}{Y})$	Log of nominal export share
log(Y)	Log of observed GDP
$\frac{IS}{Y}$	Nominal industrial supply import share
$P^{IS}$	Price of industrial supply
$\log(\frac{TB}{Y})$	Nominal trade balance share
Rest of the World	

$i^{*}$	RoW nominal Interest rate
$P^{ec,*}$	Oil price
$\log(Pop^{*})$	Log of population
$\log(P^{*})$	Log of observed GDP price
$\log(Y^{*})$	Log of observed GDP
$\log(\frac{I^{*}}{Y^{*}})$	Log of Investment to GDP
$\log(\bar{Y^{*}})$	Log of GDP trend

**Table B.2:** Selected calibrated parameters.

Monetary Policy		
Nominal interest rate in StSt	$\bar{i}$	0.004
CPI inflation in StSt	${\bar{\pi }}^{C,QA}$	0.005
Interest rate persistence	$\rho^{i}$	0.919
Response to inflation	$\eta^{i\pi }$	2.282
Response to output gap	$\eta^{iy}$	0.108
**Households**		
Preference for government bonds	$\alpha^{B}$	−0.001
Preference for stocks	$\alpha^{S}$	0.005
Preference for foreign bonds	$\alpha^{BW}$	0.013
Intertemporal discount factor	β	0.998
Savers share	$\omega^{S}$	0.670
Import share in consumption	$s^{M,C}$	0.097
Import share in investment (private and gov)	$s^{M,I}$	0.148
Import share in exports	$s^{M,X}$	0.138
Weight of disutility of labour	$\omega^{N}$	3.819
Steady-state markup	$\mu^{W}$	1.200
**Production**		
Cobb-Douglas labour share	α	0.650
Depreciation of private capital stock	δ	0.014
Share of oil in total output	$s^{IS}$	0.037
Linear capacity utilization adj. costs	$\gamma^{u,1}$	0.028
Value-added demand elasticity	$\sigma^{y}$	11.335
**Fiscal policy**		
Consumption tax	$\tau^{C}$	0.200
Corporate profit tax	$\tau^{K}$	0.300
Labour tax	$\tau^{N}$	0.438
Deficit target	$\bar{def}$	0.025
Debt target	$\bar{BG}$	3.033
**Steady-state ratios**		
Private consumption share in StSt	C/Y	0.555
Private investment share in StSt	I/Y	0.183
Govt. consumption share in StSt	$C^{G}/Y$	0.205
Govt. investment share in StSt	$I^{G}/Y$	0.031
Transfers share in StSt	T/Y	0.163
**Monetary Policy RoW**		
Interest rate persistence	$\rho^{i*}$	0.905
Response to inflation	$\eta^{i\pi *}$	1.820
Response to output gap	$\eta^{iy*}$	0.254

*Notes:* StSt refers to steady-state values.

**Table B.3:** Selected estimated parameters of exogenous shock processes.

		Prior distribution			Posterior distribution		
		Distr.	Mean	Std.	Mode	10%	90%
**EA shock processes**							
Price markup - std	$\varepsilon^{\mu }$	Gamma	0.020	0.008	0.080	0.062	0.096
Labour supply - std	$\varepsilon^{U}$	Gamma	0.010	0.004	0.007	0.005	0.011
Temporary TFP level - std	$\varepsilon^{LAY}$	Gamma	0.001	0.0004	0.0005	0.0002	0.0009
Labour demand - AR(1) coeff.	$\rho^{ND}$	Beta	0.50	0.20	0.76	0.68	0.81
Labour demand - std	$\varepsilon^{ND}$	Gamma	0.005	0.002	0.018	0.016	0.021
Government consumption - AR(1) coeff.	$\rho^{G}$	Beta	0.70	0.10	0.97	0.93	0.98
Government consumption - std	$\varepsilon^{G}$	Gamma	0.010	0.004	0.0010	0.0009	0.0011
Government investment - AR(1) coeff.	$\rho^{IG}$	Beta	0.70	0.10	0.94	0.90	0.97
Government investment - std	$\varepsilon^{IG}$	Gamma	0.010	0.004	0.0008	0.0007	0.0009
Transfers - AR(1) coeff.	$\rho^{T}$	Beta	0.70	0.10	0.95	0.88	0.98
Transfers - std	$\varepsilon^{T}$	Gamma	0.010	0.004	0.0010	0.0009	0.0011
Tax - AR(1) coeff.	$\rho^{tax}$	Beta	0.85	0.06	0.88	0.82	0.93
Tax - std	$\varepsilon^{tax}$	Gamma	0.010	0.004	0.005	0.004	0.006
Import - AR(1) coeff.	$\rho^{M,Z}$	Beta	0.45	0.15	0.87	0.85	0.89
Import - std	$\varepsilon^{M,Z}$	Gamma	0.010	0.004	0.028	0.026	0.032
Bilateral export price - AR(1) coeff.	$\rho^{X}$	Beta	0.30	0.10	0.15	0.10	0.24
Bilateral export price - std	$\varepsilon^{X}$	Gamma	0.0010	0.0004	0.0059	0.0058	0.0059
International bond preference - AR(1) coeff.	$\rho^{BW}$	Beta	0.50	0.20	0.88	0.83	0.90
International bond preference - std	$\varepsilon^{BW}$	Gamma	0.010	0.004	0.0028	0.0024	0.0043
Monetary policy - std	$\varepsilon^{i}$	Gamma	0.010	0.004	0.0009	0.0008	0.0011
**RoW shock processes**							
Time preference - AR(1) coeff.	$\rho^{C,*}$	Beta	0.01	0.00	0.01	0.01	0.02
Time preference - std	$\varepsilon^{C,*}$	Gamma	0.50	0.20	0.82	0.72	0.86
Investment risk prem. - AR(1) coeff.	$\rho^{S,*}$	Beta	0.01	0.00	0.01	0.01	0.02
Investment risk prem. - std	$\varepsilon^{S,*}$	Gamma	0.50	0.20	0.86	0.81	0.91
Price markup	$\varepsilon^{\mu ,*}$	Gamma	0.010	0.004	0.039	0.010	0.059
Import - AR(1) coeff.	$\rho^{M,Z*}$	Beta	0.45	0.15	0.86	0.83	0.89
Import - std	$\varepsilon^{M,Z*}$	Gamma	0.010	0.004	0.030	0.026	0.033
Monetary policy - std	$\varepsilon^{i,*}$	Gamma	0.010	0.004	0.0019	0.0016	0.0023

**Table C.1:** Fit. 1-quarter ahead.

	base (est. 19q4)	base (est. 21q4)	FS	DF	FS + DF
**2000q1 : 2021q4**					
GDP growth	−0.23	−0.14	−0.02	0.10	0.40
Consumption growth	−0.68	−0.15	−0.79	−0.19	0.50
Hours growth	−0.40	−0.15	0.09	0.14	0.42
Inflation (consumption deflator)	0.16	−0.01	0.16	0.16	0.18
**2000q1 : 2019q4**					
GDP growth	−0.10	−0.18	−0.23	−0.34	−0.29
Consumption growth	0.18	−0.08	0.31	0.16	0.28
Hours growth	0.28	−0.43	−0.33	−0.35	−0.45
Inflation (consumption deflator)	0.22	0.13	0.15	0.11	0.13

*Notes:* Columns display the $R^{2}$ defined as 1 minus the ratio of the 1-step-ahead (one quarter) forecast error to the deviation of the observed time series from the model-implied steady state. This definition implies that our $R^{2}$ has an upper bound at 1 (no forecast errors) and is unbounded from below (the volatility of the forecast error can exceed the volatility of the time series). $R^{2}$ declines monotonically as the forecast error increases. All variables are expressed in growth rates and real terms (except inflation, which is a growth rate itself and a nominal variable). Consumption refers to private consumption. The first two columns report results of the baseline GM model without COVID-specific shocks estimated using data until 2019q4 and 2021q4, respectively. Columns ‘FS’ and ‘DF’ report results from our benchmark model with ‘forced savings’ and from the MA(1)-discount factor shock specification outlined in Section 9.2 of the main text. The last column shows results from the mixed model (‘FS + DF’).

**Table C.2:** Fit. 1-year ahead.

	base (est. 19q4)	base (est. 21q4)	FS	DF	FS + DF
**2000q1 : 2021q4**					
GDP growth	0.54	0.47	0.47	0.57	0.55
Consumption growth	0.04	0.10	−0.47	0.17	0.15
Hours growth	0.41	0.35	0.47	0.54	0.51
Inflation (consumption deflator)	0.58	0.63	0.63	0.60	0.59
**2000q1 : 2019q4**					
GDP growth	0.63	0.56	0.61	0.57	0.57
Consumption growth	0.79	0.49	0.76	0.71	0.74
Hours growth	0.71	0.47	0.61	0.55	0.56
Inflation (consumption deflator)	0.66	0.61	0.63	0.58	0.58

*Notes:* Columns display the $R^{2}$ as 1 minus the ratio of the error of the annualised forecast to the deviation of the (annualised) observed time series from the model-implied steady state. See also the notes below Table C.1.

**Table C.3:** Comparison of selected point estimates.

		base (est. 19q4)	base (est. 21q4)	FS	DF	FS + DF
**Nominal and real frictions**						
Consumption habit persistence	h	0.88	0.19	0.89	0.66	0.83
Inverse Frisch elasticity	$\theta^{N}$	3.12	2.66	2.45	2.28	2.39
Employment adjustment cost	$\gamma^{N}$	2.54	0.05	0.02	0.24	0.02
Investment adjustment cost	$\gamma^{I,2}$	216.14	126.31	48.52	68.76	36.61
Real wage rigidity	$\gamma^{wr}$	0.93	1.00	0.92	0.91	0.92
**Shock processes**						
Discount factor - AR(1) coeff.	$\rho^{P}$	0.74	0.40	0.74	0.78	0.72
Discount factor - std. (%)	$\varepsilon^{P}$	0.009	0.007	0.010	0.004	0.011
**COVID related shocks**						
Forced saving shock	$\varepsilon^{tC}$	-	-	0.043	-	0.038
Discount factor - MA(1) coeff.	$\rho^{DF}$	-	-	-	0.99	0.99
Discount factor - std. (%)	$\varepsilon^{DF}$	-	-	-	0.098	0.099

*Notes:* The first two columns report results from the baseline model without COVID-specific shocks, estimated using data until 2019q4 and 2021q4, respectively. Columns ‘FS’ and ‘DF’ report results from our benchmark model with ‘forced savings’ and MA(1)-discount factor shock specification outlined in Section 9.2. The last column shows results from the mixed model (‘FS + DF’).

## Appendix A Model details

This appendix provides additional model details omitted in the main text. The model shares many standard elements with Albonico et al. (2019), and we also refer to the model description contained therein. Section 6.1

### A1 Households

There are two representative households indexed s and c, respectively. Savers, s, maximise lifetime utility

(A.1) $$E_{0}\sum_{t=0}^{\infty }\beta^{t}\xi_{t}\left\{\frac{{\left(C_{j,t}^{s}-\varepsilon_{t}^{\mathrm{tC}}-h\left(C_{t-1}^{s}-\varepsilon_{t-1}^{\mathrm{tC}}\right)\right)}^{1-\theta }}{1-\theta }-\omega_{t}^{N}\frac{{\left(N_{j,t}^{s}\right)}^{1+\theta^{N}}}{1+\theta^{N}}-\frac{{\bar{\lambda^{s}}}_{t}}{P_{t}^{C}}\sum_{Q}B_{j,t-1}^{Q}\left(\alpha^{Q}-\varepsilon_{t-1}^{Q}\right)\right\},$$

subject to a sequence of budget constraints

(A.2) $$P_{t}^{C}C_{j,t}^{s}+\sum_{Q}B_{j,t}^{Q}=W_{t}\left(N_{j,t}^{s}+\varepsilon_{t}^{\mathrm{tN}}\right)+D_{t}+\sum_{Q}R_{t-1}^{Q}B_{j,t-1}^{Q}+T_{j,t}^{s},$$

we define $\xi_{t+1}/\xi_{t}=\exp\left(\varepsilon_{t}^{C}\right)$, where $\varepsilon_{t}^{C}$ is an exogenous process consisting of a relatively short-lived (estimated) and a calibrated highly persistent component with an autoregressive coefficient of 0.975,^28^ $\varepsilon_{t}^{tC}$ is the non-persistent ‘forced-savings’ shocks, and $\varepsilon_{t}^{N}$ represents a temporary labour hoarding shock. The portfolio consists of risk-free domestic bonds (rf), one internationally traded asset (bw), denoted in RoW currency, and domestic firm shares (S). We also include government bonds (G) in the portfolio. Each asset has a gross nominal return $R_{t}^{Q}$ (bond returns are pre-determined, whereas the return on firm shares is unknown at time t). The net of transfers and taxes is

(A.3) $$T_{j,t}^{s}=TR_{j,t}^{s}-\mathrm{ta}x_{j,t}^{s}-\tau^{N}W_{t}\left(N_{j,t}^{s}+\varepsilon_{t}^{\mathrm{tN}}\right)-\tau^{C}P_{t}^{C}C_{j,t}^{s},$$

where $TR_{j,t}^{s},tax_{j,t}^{s}$, $\tau^{C}$ and $\tau^{N}$ denote transfers, lump-sum taxes, the consumption (sales) tax and the labour tax rate, respectively. Transfers are partly endogenised with unemployment benefits, $BEN_{j,t}^{s}$ which are computed as the gap between actual and potential hours^29^ times the unemployment benefit replacement rate, $\tau^{u}$:

(A.4) $$TR_{j,t}^{s}=BEN_{j,t}^{s}+\omega^{s}P_{t}T_{t},$$

(A.5) $$\mathrm{BE}N_{j,t}^{s}=\tau^{u}W_{t}\left(N_{t}^{\mathrm{pot}}-\left(N_{j,t}^{s}+\varepsilon_{t}^{\mathrm{tN}}\right)\right).$$

The residual component of transfers, $P_{t}T_{t}$ follows the fiscal rule described in A.4.

The first order necessary conditions in a symmetric equilibrium are for Q∈{rf,bw,S,G}:

(A.6) $$1=E_{t}[\Lambda_{t,t+1}^{s}\frac{R_{t}^{Q}+\varepsilon_{t}^{Q}-\alpha^{Q}}{1+\pi_{t+1}^{C,vat}}],$$

where $\alpha^{rf}=0$, $\lambda_{t}^{s}={\left(C_{t}^{s}-h^{s}C_{t-1}^{s}\right)}^{-\theta }$, and $\Lambda_{t,t+1}^{s}=\beta \exp\left(\varepsilon_{t}^{C}\right)\frac{\lambda_{t+1}^{s}}{\lambda_{t}^{s}}$.

Approximating the first-order condition for investment in foreign bonds gives a standard uncovered interest rate parity condition:

(A.7) $$E_{t}[\frac{E_{t+1}}{E_{t}}]i_{t}^{W}=i_{t}^{rf}+rprem_{t}^{W},$$

where $i_{t}^{W}$ and $rprem_{t}^{W}$ are the return and risk premium on the foreign bond, respectively.

The remaining households with the population share $1-\omega^{s}$ are liquidity-constrained (c). In each period, they consume their wage incomes and net transfers/taxes^30^. During the pandemic, households may accumulate ‘forced savings’ that will be spent gradually over time:

(A.8) $$P_{t}^{C}C_{j,t}^{c}=W_{t}\left(N_{j,t}^{c}+\varepsilon_{t}^{tN}\right)+T_{j,t}^{c}+P_{t}^{C}(\varepsilon_{t}^{tC}-\frac{1}{6}\sum_{i=8}^{13}\varepsilon_{t-i}^{tC}).$$

Total consumption by EA households is

(A.9) $$C_{t}=\left(1-\omega^{s}\right)C_{t}^{c}+\omega^{s}C_{t}^{s}$$

and total EA labour supply

(A.10) $$N_{t}=\left(1-\omega^{s}\right)N_{t}^{c}+\omega^{s}N_{t}^{s}.$$

### A2 Wage setting

The labour market structure follows Albonico et al. (2019): A labour union sells labour services $N_{j,t}$ provided by both types of domestic households to intermediate good producing firms. We assume that Ricardian and liquidity-constrained households’ hours are distributed proportionally to their respective population shares. Since both households face the same labour demand schedule, each household works the same number of hours as the economy’s average. It follows that the individual union’s choice variable is a common nominal wage rate for both types of households. The union maximises the discounted future stream of the weighted average of lifetime utility of its members with respect to the wage, and it is subject to the weighted sum of their budget constraints:

(A.11) $$\max_{W_{j,t}}U_{j,t}=\sum_{t=0}^{\infty }\beta^{t}\xi_{t}U\left(C_{j,t},N_{j,t},\cdot \right)$$

subject to:

(A.12) $$P_{t}^{C}C_{j,t}+\omega^{s}\sum_{Q}B_{j,t}^{Q}+\Gamma_{\mathrm{jt}}^{W}=\left(1-\tau^{N}\right)W_{j,t}\left(N_{j,t}+\varepsilon_{t}^{\mathrm{tN}}\right)+\omega^{s}\left(\sum_{Q}R_{t-1}^{Q}B_{j,t-1}^{Q}+D_{t}\right)+T_{j,t}$$

(A.13) $$\begin{array}{cc} \;\\ N_{j,t} & =(\frac{W_{j,t}}{W_{t}}){}^{-\sigma^{n}}N_{t}, \end{array}$$

where $\Gamma_{j,t}^{W}\equiv \frac{\gamma^{w}\left(\sigma^{n}-1\right)}{2}W_{t}N_{t}{\left(\pi_{t}^{w}-\pi^{w}-\left(1-\mathrm{sfw}\right)\left(\pi_{t-1}^{C,\mathrm{vat}}-\bar{\pi }\right)\right)}^{2}$ is a quadratic wage adjustment cost that is born by the households and 1-sfw is the share of wage setters that index the growth rate of wages to the previous period inflation. $\sigma^{n}$ is the inverse of the steady-state gross wage markup. Additionally, we allow for a slow adjustment of real wages as in Blanchard and Galí (2007). The resulting wage equation is:

(A.14) $$\mu^{w}{\left(\frac{U_{N,t}}{\lambda_{t}}-\tau^{u}\frac{W_{t}}{P_{t}^{C,\mathrm{vat}}}-\mu_{t}^{w}\right)}^{1-\gamma^{\mathrm{wr}}}{\left[\frac{\left(1-\tau^{N}\right)W_{t-1}}{P_{t-1}^{C,\mathrm{vat}}}\right]}^{\gamma^{\mathrm{wr}}}=\frac{W_{t}}{P_{t}^{C,\mathrm{vat}}}\left(1-\tau^{N}\right),$$

where $\mu_{t}^{w}$ is the fluctuating gross wage markup:

$$\mu_{t}^{w}=\gamma^{w}\left[\frac{\partial \Gamma_{t}^{w}}{\partial W_{t}}-\beta_{t}E_{t}\frac{\lambda_{t+1}}{\lambda_{t}}\frac{1}{\pi_{t+1}^{C,\mathrm{vat}}+1}\frac{\partial \Gamma_{t+1}^{w}}{\partial W_{t}}\right].$$

$\mu^{w}={\left(\frac{\sigma^{n}}{1-\sigma^{n}}\right)}^{\gamma^{wr}-1}$ is the steady-state markup, $\gamma^{wr}$ and $\gamma^{w}$ govern real and nominal rigidity, respectively. $\varepsilon_{t}^{U}$ is a labour supply shock. $U_{N,t}$ is the derivative of the utility function with respect to labour. $P_{t}^{C,vat}$ is the price of consumption goods adjusted for the sales tax ($P_{t}^{C,vat}=\left(1+\tau^{C}\right)P_{t}^{C}$).

### A3 Intermediate goods

Each firm i∈[0,1] produces a variety of domestic goods, which is an imperfect substitute for varieties produced by other firms. Firms are monopolistically competitive and face a downward-sloping demand function for goods.

Differentiated goods are produced using private and government capital, $K_{i,t-1},K_{t-1}^{G}$, and labour, $N_{i,t}$ net of a constant overhead component $FN_{i}$, which are combined in a Cobb-Douglas production function:

(A.15) $$Y_{i,t}={\left[A_{t}^{Y}\left(N_{i,t}-FN_{i}\right)\right]}^{\alpha }{\left(cu_{i,t}K_{i,t-1}\right)}^{1-\alpha }{\left(K_{t-1}^{G}\right)}^{1-\alpha_{G}}-A_{t}^{Y}\Phi ,$$

where α is the steady-state labour share, $A_{t}^{Y}$ represents the labour-augmenting productivity common to all firms in the differentiated goods sector, $cu_{i,t}$ denotes firm-specific capital utilization. Φ captures fixed costs in production. Since total factor productivity (TFP) is not a stationary process, we allow for two types of technology shocks, $\varepsilon_{t}^{GA}$ and $\varepsilon_{t}^{A}$. They are related to a non-stationary process and its autoregressive component $\rho^{A}$:

(A.16) $$\log\left(A_{t}^{Y}\right)-\log\left(A_{t-1}^{Y}\right)=g_{t}^{A}+\varepsilon_{t}^{A},$$

(A.17) $$g_{t}^{A}=\rho^{A}g_{t-1}^{A}+\left(1-\rho^{A}\right)g^{A}+\varepsilon_{t}^{GA},$$

where $g_{t}^{A}$ and $g^{A}$ are the time-varying growth and the long-run growth of technology, respectively.

Monopolistically competitive firms maximize the real value of the firm $\frac{P_{t}^{S}}{P_{t}}S_{t}^{tot}$, that is the discounted stream of expected future profits, subject to the output demand $Y_{i,t}={\left(\frac{P_{i,t}}{P_{t}}\right)}^{-\sigma^{y}}Y_{t}$, the technology constraint (A.15), and a capital accumulation equation $K_{i,t}=I_{i,t}+\left(1-\delta \right)K_{i,t-1}$.^31^ Their problem can be written as:

(A.18) $$\max_{P_{i,t},N_{i,t},I_{i,t},cu_{i,t},K_{i,t}}\sum_{s=t}^{\infty }D^{S}\Pi_{i,t}^{f},$$

where the stochastic discount factor, $D^{S}$, is:

(A.19) $$D^{S}=\frac{1+r_{t}^{S}}{\Pi_{r=t}^{S}\left(1+r_{r}^{S}\right)}$$

with $1+r_{t}^{S}=\frac{1+i_{t+1}^{S}}{1+\pi_{t+1}}$ being the real stock return.

$P_{i,t}$ is the price of intermediate inputs, and the corresponding price index is:

(A.20) $$P_{t}=(\int_{0}^{1}{\left(P_{i,t}\right)}^{1-\sigma^{y}}di){}^{\frac{1}{1-\sigma^{y}}}.$$

The period t profit of an intermediate goods firm i is given by:

(A.21) $$\Pi_{i,t}^{f}=\left(1-\tau^{K}\right)(\frac{P_{i,t}}{P_{t}}Y_{i,t}-\frac{W_{t}}{P_{t}}\left(N_{i,t}+\varepsilon_{t}^{tN}\right))+\tau^{K}\delta \frac{P_{t}^{I}}{P_{t}}K_{i,t-1}-\frac{P_{t}^{I}}{P_{t}}I_{i,t}-\Gamma_{i,t},$$

where $I_{i,t}$ is the physical investment at price $P_{i,t}^{I}$, $\tau^{K}$ is the corporate tax rate and δ the capital depreciation rate.

Firms face quadratic factor adjustment costs, $\Gamma_{i,t}$, measured in terms of production input factors:

(A.22) $$\Gamma_{i,t}=\Gamma_{i,t}^{P}+\Gamma_{i,t}^{N}+\Gamma_{i,t}^{I}+\Gamma_{i,t}^{cu}.$$

Specifically, the adjustment costs are associated with the output price $P_{i,t}$, labour input $N_{i,t}$, investment $I_{i,t}$, as well as capacity utilisation $cu_{i,t}$ variation:

(A.23) $$\Gamma_{i,t}^{P}\equiv \sigma^{y}\frac{\gamma^{P}}{2}Y_{t}{\left[\frac{P_{i,t}}{P_{i,t-1}}-\exp\left(\bar{\pi }\right)\right]}^{2},$$

(A.24) $$\Gamma_{i,t}^{N}\equiv \frac{\gamma^{N}}{2}Y_{t}{\left[\frac{N_{i,t}+\varepsilon_{t}^{\mathrm{tN}}}{N_{i,t-1}+\varepsilon_{t-1}^{\mathrm{tN}}}-\exp\left(g^{\mathrm{pop}}\right)\right]}^{2},$$

(A.25) $$\Gamma_{i,t}^{I}\equiv \frac{P_{t}^{I}}{P_{t}}\left[\frac{\gamma^{I,1}}{2}K_{t-1}{\left(\frac{I_{i,t}}{K_{t-1}}-\delta_{t}^{K}\right)}^{2}+\frac{\gamma^{I,2}}{2}\frac{{\left(I_{i,t}-I_{i,t-1}\exp\left(g^{Y}+g^{P^{I}}\right)\right)}^{2}}{K_{t-1}}\right],$$

(A.26) $$\Gamma_{i,t}^{\mathrm{cu}}\equiv \frac{P_{t}^{I}}{P_{t}}K_{i,t-1}^{\mathrm{tot}}\left[\gamma^{\mathrm{cu},1}\left(cu_{i,t}-1\right)+\frac{\gamma^{\mathrm{cu},2}}{2}{\left(cu_{i,t}-1\right)}^{2}\right],$$

where γ-parameters capture the degree of adjustment costs. $\bar{\pi }$ denotes steady-state inflation. $g^{pop}$, $g^{Y}$, and $g^{PI}$ are trend factors of population, GDP and prices for investment goods, respectively. $\delta_{t}^{K}\neq \delta$ is a function of the depreciation rate adjusted for the capital trend in order to have zero adjustment costs on the trend path.^32^

Given the Lagrange multiplier associated with the technology constraint, $\mu^{y}$, the FOCs with respect to labour, capital, investments and capital utilisation are given by:

(A.27) $$\left(1-\tau^{K}\right)\frac{W_{t}}{P_{t}}=\alpha \left(\mu_{t}^{y}-\varepsilon_{t}^{ND}\right)\frac{Y_{t}}{N_{t}}-\frac{\partial \Gamma_{t}^{N}}{\partial N_{t}}+E_{t}[\frac{1+\pi_{t+1}}{1+i_{t+1}^{s}}\frac{\partial \Gamma_{t+1}^{N}}{\partial N_{t}}],$$

(A.28) $$Q_{t}=E_{t}\left[\frac{1+\pi_{t+1}}{1+i_{t+1}^{s}}\frac{P_{t+1}^{I}}{P_{t+1}}\frac{P_{t}}{P_{t}^{I}}\left(\tau^{K}\delta^{K}-\frac{\partial \Gamma_{t}^{\mathrm{cu}}}{\partial K_{t-1}}+Q_{t+1}\left(1-\delta^{K}\right)+\left(1-\alpha \right)\mu_{t+1}^{y}\frac{P_{t+1}}{P_{t+1}^{I}}\frac{Y_{t+1}}{K_{t}}\right)\right],$$

(A.29) $$\begin{array}{c} \frac{I_{t}}{K_{t-1}}-\delta_{t}^{K}=sli_{t}(\zeta_{1}\left(\frac{GOS_{t}}{K_{t-1}}\frac{P_{t}}{P_{t}^{I}}\right)-\zeta_{0}-\delta^{K})+\frac{1-sli_{t}}{\gamma^{I,1}}Q_{t}-1-\gamma^{I,2}\frac{\left(I_{t}-I_{t-1}\exp(g^{Y}+g^{P^{I}})\right)}{K_{t-1}}\\ +E_{t}[\frac{1+\pi_{t+1}}{1+i_{t+1}^{s}}\frac{P_{t+1}^{I}}{P_{t+1}}\frac{P_{t}}{P_{t}^{I}}\exp(g^{Y}+g^{P^{I}})\gamma^{I,2}\frac{\left(I_{t+1}-I_{t}\exp(g^{Y}+g^{P^{I}})\right)}{K_{t}}], \end{array}$$

(A.30) $$\mu_{t}^{y}\left(1-\alpha \right)\frac{Y_{t}}{cu_{t}}\frac{P_{t}}{P_{t}^{I}}=K_{t-1}^{tot}[\gamma^{u,1}+\gamma^{u,2}\left(cu_{t}-1\right)],$$

where $Q_{t}=\mu_{t}^{y}/\frac{P_{t}^{I}}{P_{t}}$.

In a symmetric equilibrium ($P_{i,t}=P_{t}$), the FOC with respect to $P_{i,t}$ yields the New Keynesian Phillips curve:

(A.31) $$\begin{array}{cc} \mu_{t}^{y}\sigma^{y} & =\left(1-\tau^{K}\right)\left(\sigma^{y}-1\right)+\sigma^{y}\gamma^{P}\frac{P_{t}}{P_{t-1}}(\pi_{t}-\bar{\pi })\\ & -\sigma^{y}\gamma^{P}[\frac{1+\pi_{t+1}}{1+i_{t+1}^{s}}\frac{P_{t+1}}{P_{t}}\frac{Y_{t+1}}{Y_{t}}(\pi_{t+1}-\bar{\pi })]+\sigma^{y}\varepsilon_{t}^{\mu }, \end{array}$$

where here $\varepsilon_{t}^{\mu }$ is a white noise markup shock. The final New Keynesian Phillips curve takes then the following form:

(A.32) $$\mu_{t}^{y}\sigma^{y}=\left(1-\tau^{K}\right)\left(\sigma^{y}-1\right)+\sigma^{y}\gamma^{P}\frac{P_{t}}{P_{t-1}}(\pi_{t}-\bar{\pi })-\sigma^{y}\gamma^{P}\frac{1+\pi_{t+1}}{1+i_{t+1}^{s}}\frac{P_{t+1}}{P_{t}}\frac{Y_{t+1}}{Y_{t}}[sfp(\pi_{t+1}-\bar{\pi })+\left(1-sfp\right)\left(\pi_{t-1}-\bar{\pi }\right)]+\sigma^{y}\varepsilon_{t}^{\mu },$$

where sfp is the share of forward-looking price setters.

### A4 Fiscal policy

The government collects taxes on labour, $\tau^{N}$, capital, $\tau^{K}$, consumption, $\tau^{C}$, and lump-sum taxes, $tax_{t}$, and issues one-period bonds, $B_{t}^{G}$, to finance government consumption, $G_{t}$, investment, $I_{t}^{G}$, transfers, $T_{t}$, and the servicing of the outstanding debt. The tax on commodity imports from RoW, $\tau^{IS}$, is fixed. The government budget constraint is:

(A.33) $$B_{t}^{G}=\left(1+i_{t-1}^{G}\right)B_{t-1}^{G}-R_{t}^{G}+P_{t}^{G}G_{t}+P_{t}^{IG}I_{t}^{G}+T_{t}P_{t},$$

where nominal government revenues, $R^{G}$, are defined as:

(A.34) $$\begin{array}{cc} R_{t}^{G} & \equiv \tau^{K}\left(P_{t}Y_{t}-W_{t}\left(N_{t}+\varepsilon_{t}^{\mathrm{tN}}\right)-P_{t}^{I}\delta K_{t-1}\right)+\tau^{N}W_{t}\left(N_{t}+\varepsilon_{t}^{\mathrm{tN}}\right)+\tau^{C}P_{t}^{C}C_{t}\\ & +\tau^{\mathrm{IS}}P_{t}^{Y0}IS_{t}+\mathrm{ta}x_{t}P_{t}Y_{t}. \end{array}$$

The government closes its budget via lump sum taxes:

(A.35) $$tax_{t}=\rho^{\tau }tax_{t-1}+\eta^{d}\left(\frac{\Delta B_{t-1}^{G}}{Y_{t-1}P_{t-1}}-\bar{def}\right)+\eta^{B}\left(\frac{B_{t-1}^{G}}{Y_{t-1}P_{t-1}}-\bar{BG}\right)+\varepsilon_{t}^{tax},$$

where $\bar{def}$ and $\bar{BG}$ are the targets on government deficit and government debt with debt rule coefficients $\eta^{d}$ and $\eta^{B}$, respectively. $\varepsilon_{t}^{tax}$ is a white noise shock. $\rho^{\tau }$ governs the debt rule persistence.

The accumulation equation for government capital is:

(A.36) $$K_{t}^{G}=\left(1-\delta \right)K_{t-1}^{G}+I_{t}^{G},$$

where δ is the depreciation rate.

The model uses a measure of discretionary fiscal effort (DFE) as defined by the European Commission (2013):

(A.37) $$\mathrm{DF}E_{t}\equiv \frac{R_{t}^{G}}{Y_{t}^{N}}-\frac{\Delta E_{t}^{G}-\left(\frac{y_{t}^{\mathrm{pot}}}{y_{t-1}^{\mathrm{pot}}}-1\right)E_{t-1}^{G}}{Y_{t}},$$

where $R_{t}^{G}$ stands for government revenues in nominal terms, $E_{t}^{G}$ is the adjusted nominal expenditure aggregate, $y_{t}^{pot}$ is the medium-term nominal potential output, and $Y_{t}^{N}$ is nominal GDP.^33^ Following the definition of DFE, we define the aggregate nominal expenditure as:

(A.38) $$E_{t}^{G}\equiv P_{t}^{G}G_{t}+P_{t}^{\mathrm{IG}}I_{t}^{G}+P_{t}T_{t}.$$

We use the following discretionary fiscal effort rules for government consumption, $G_{t}$, investment, $I_{t}^{G}$, and transfers, $T_{t}$:

(A.39) $$\frac{G_{t}P_{t}^{G}-G_{t-1}P_{t-1}^{G}}{P_{t}Y_{t}}=(\frac{Y_{t}^{pot}}{Y_{t-1}^{pot}}\exp\left(\pi_{t}\right)-1)\frac{G_{t-1}P_{t-1}^{G}}{P_{t}Y_{t}}+\varepsilon_{t}^{G}-\varepsilon_{t-1}^{G},$$

(A.40) $$\frac{I_{t}^{G}P_{t}^{IG}-I_{t-1}^{G}P_{t-1}^{IG}}{P_{t}Y_{t}}=(\frac{Y_{t}^{pot}}{Y_{t-1}^{pot}}\exp\left(\pi_{t}\right)-1)\frac{I_{t-1}^{G}P_{t-1}^{IG}}{P_{t}Y_{t}}+\varepsilon_{t}^{IG}-\varepsilon_{t-1}^{IG},$$

(A.41) $$\frac{T_{t}P_{t}-T_{t-1}P_{t-1}}{P_{t}Y_{t}}=(\frac{Y_{t}^{pot}}{Y_{t-1}^{pot}}\exp\left(\pi_{t}\right)-1)\frac{T_{t-1}P_{t-1}}{P_{t}Y_{t}}+\varepsilon_{t}^{T}-\varepsilon_{t-1}^{T},$$

where $\varepsilon_{t}^{G}$, $\varepsilon_{t}^{IG}$, $\varepsilon_{t}^{T}$ are shocks to government consumption, investment and transfers, respectively.

### A5 RoW details

The intermediate good producers use labour to manufacture domestic goods (non-oil output) according to a production function:

(A.42) $$Y_{i,t}^{*}={\left(A_{t}^{Y^{*}}N_{i,t}^{*}\right)}^{\alpha^{*}}{\left(cu_{i,t}^{*}K_{i,t-1}^{*}\right)}^{1-\alpha^{*}},$$

where $A_{t}^{Y,*}$ captures a trend in the productivity, $N_{t}^{*}$ is total hours, $K_{i,t-1}^{*}$ (following a similar law of motion as capital in the EA) and $cu_{i,t}^{*}$ is beginning of period capital and its utilization intensity, respectively.

Given the Lagrange multiplier associated with the technology constraint, $\mu^{y^{*}}$, price setting for non-oil output follows a New Keynesian Phillips curve:

(A.43) $$\begin{array}{ccc} \mu_{t}^{y^{*}}\sigma^{y^{*}} & = & \left(\sigma^{y^{*}}-1\right)+\sigma^{y^{*}}\gamma^{P^{*}}\frac{P_{t}^{*}}{P_{t-1}^{*}}\left(\pi_{t}^{*}-\bar{\pi^{*}}\right)\\ & & -\;\sigma^{y^{*}}\gamma^{P^{*}}\frac{1+\pi_{t+1}^{*}}{1+i_{t+1}^{s^{*}}}\frac{{P^{*}}_{t+1}}{P_{t}^{*}}\frac{{Y^{*}}_{t+1}}{Y_{t}^{*}}\left[\mathrm{sf}p^{*}\left(\pi_{t+1}^{*}-\bar{\pi^{*}}\right)+\left(1-\mathrm{sf}p^{*}\right)\left(\pi_{t-1}^{*}-\bar{\pi^{*}}\right)\right]+\sigma^{y^{*}}\varepsilon_{t}^{\mu^{*}}, \end{array}$$

where $\varepsilon_{t}^{\mu^{*}}$ is a cost-push shock, and $sfp^{*}$ is the share of forward-looking price setters.

Optimality conditions for labour, capital, investment and capital utilisation are:

(A.44) $$\frac{W_{t}^{*}}{P_{t}^{*}}=\alpha \left(\mu_{t}^{y^{*}}-\varepsilon_{t}^{ND^{*}}\right)\frac{Y_{t}^{*}}{N_{t}^{*}}-\frac{\partial \Gamma_{t}^{N^{*}}}{\partial N_{t}^{*}}+E_{t}\left[\frac{1+\pi_{t+1}^{*}}{1+i_{t+1}^{s^{*}}}\frac{\partial \Gamma_{t+1}^{N^{*}}}{\partial N_{t}^{*}}\right],$$

(A.45) $$Q_{t}^{*}=E_{t}\left[\frac{1+\pi_{t+1}^{*}}{1+i_{t+1}^{s^{*}}}\frac{P_{t+1}^{I^{*}}}{P_{t+1}^{*}}\frac{P_{t}^{*}}{P_{t}^{I^{*}}}\left(-\frac{\partial \Gamma_{t}^{cu^{*}}}{\partial K_{t-1}^{*}}+Q_{t+1}^{*}\left(1-\delta^{K*}\right)+\left(1-\alpha \right)\mu_{t+1}^{y^{*}}\frac{P_{t+1}^{*}}{P_{t+1}^{I^{*}}}\frac{Y_{t+1}^{*}}{K_{t}^{*}}\right)\right],$$

(A.46) $$\begin{array}{ccc} \frac{I_{t}^{*}}{K_{t-1}^{*}}-\delta_{t}^{{}^{*}} & = & \mathrm{sl}i_{t}^{*}\left(\zeta_{1}^{*}\left(\frac{\mathrm{GO}S_{t}^{*}}{K_{t-1}^{*}}\frac{P_{t}^{*}}{P_{t}^{I^{*}}}\right)-\zeta_{0}^{*}-\delta^{K*}\right)+\frac{1-\mathrm{sl}i_{t}^{*}}{\gamma^{I^{*},1}}Q_{t}^{*}-1-\gamma^{I^{*},2}\frac{\left(I_{t}^{*}-I_{t-1}^{*}\exp\left(g^{Y}+g^{P^{I^{*}}}\right)\right)}{K_{t-1}^{*}}\\ & & +\;E_{t}\left[\frac{1+\pi_{t+1}^{*}}{1+i_{t+1}^{s^{*}}}\frac{P_{t+1}^{I^{*}}}{P_{t+1}^{*}}\frac{P_{t}^{*}}{P_{t}^{I^{*}}}\exp\left(g^{Y}+g^{P^{I^{*}}}\right)\gamma^{I^{*},2}\frac{\left(I_{t+1}^{*}-I_{t}^{*}\exp\left(g^{Y}+g^{P^{I^{*}}}\right)\right)}{K_{t}^{*}}\right], \end{array}$$

(A.47) $$\mu_{t}^{y^{*}}\left(1-\alpha \right)\frac{Y_{t}^{*}}{cu_{t}^{*}}\frac{P_{t}^{*}}{P_{t}^{I^{*}}}=K_{t-1}^{*}\left[\gamma^{u^{*},1}+\gamma^{u^{*},2}\left(cu_{t}^{*}-1\right)\right],$$

where $Q_{t}^{*}=\mu_{t}^{y^{*}}/\frac{P_{t}^{I^{*}}}{P_{t}^{*}}$.

*Commodities*. The price of the commodity bundle is

(A.48) $$P_{t}^{IS^{*}}=\varepsilon_{t}^{pIS,*}[\left(1-s^{ec}\right){\left(P_{t}^{nec^{*}}\right)}^{1-\sigma^{IS}}+s^{ec}{\left(P_{t}^{ec^{*}}\right)}^{1-\sigma^{IS}}]{}^{\frac{1}{1-\sigma^{IS}}},$$

with substitution elasticity $\sigma^{IS}$, share parameter $s^{ec}$, and commodity bundle price shock $\varepsilon_{t}^{pIS,*}$.

*RoW Households*. RoW households maximise a utility function similar to EA Ricardian households, subject to the budget constraint:

(A.49) $$P_{t}^{C^{*}}C_{j,t}^{*}+BW_{j,t+1}^{*}=W_{t}^{*}N_{j,t}^{*}+D_{t}^{*}+R_{t}^{BW^{*}}BW_{j,t}^{*},$$

where assets $Q^{*}$ include only RoW firm shares and the international traded asset. We allow the RoW savings preferences to be affected by the non-persistent ‘forced savings’ shocks, $\epsilon_{t}^{tc^{*}}$. However, we abstract from the temporary labour hoarding shock in the RoW due to data limitations (no quarterly data available for the RoW).

The first order conditions of the household problem read:

(A.50) $$1=E_{t}[\Lambda_{t,t+1}^{*}\frac{R_{t}^{Q^{*}}+\varepsilon_{t}^{Q^{*}}-\alpha^{Q^{*}}}{1+\pi_{t+1}^{C^{*}}}],$$

where $\alpha^{rf^{*}}=0$, $\lambda_{t}^{*}={\left(C_{t}^{*}-h^{*}C_{t-1}^{*}\right)}^{-\theta^{*}}$, and $\Lambda_{t,t+1}^{*}=\beta^{*}\exp\left(\varepsilon_{t}^{C^{*}}\right)\frac{\lambda_{t+1}^{*}}{\lambda_{t}^{*}}$. Wage setting in RoW follows the description in A.2 with the only differences being the absence of the labour tax and the presence of only one representative household. This translates into the following wage equation:

(A.51) $$(\frac{U_{N^{*},t}}{\lambda_{t}^{*}}){}^{1-\gamma^{wr^{*}}}{\left[W_{t-1}^{*}P_{t-1}^{C^{*}}\right]}^{\gamma^{wr^{*}}}=\frac{W_{t}^{*}}{P_{t}^{C^{*}}}\mu_{t}^{w^{*}},$$

where $\mu_{t}^{w^{*}}$ is the fluctuating gross wage markup:

$$\mu_{t}^{w^{*}}=\mu^{w^{*}}+\mu^{w^{*}}\gamma^{w^{*}}\left[\frac{\partial \Gamma_{t}^{w^{*}}}{\partial W_{t}^{*}}-\beta_{t}^{*}E_{t}\frac{\lambda_{t+1}^{*}}{\lambda_{t}^{*}}\frac{1}{\pi_{t+1}^{C^{*}}+1}\frac{\partial \Gamma_{t+1}^{w^{*}}}{\partial W_{t}^{*}}+\frac{1}{\gamma^{w}}\varepsilon_{t}^{U^{*}}\right],$$

and $\mu^{w^{*}}={\left(\frac{\sigma^{n^{*}}}{1-\sigma^{n^{*}}}\right)}^{\gamma^{wr^{*}}-1}$ is the steady-state markup. $\gamma^{wr^{*}}$ and $\gamma^{w^{*}}$ govern real and nominal rigidity, respectively. $\varepsilon_{t}^{U^{*}}$ is a labour supply shock. $U_{N^{*}}$ is the derivative of the utility function with respect to labour.

The aggregate resource constraint in the RoW is:

(A.52) $$P_{t}^{*}Y_{t}^{*}+P_{t}^{I,*}I_{t}^{*}=P_{t}^{C,*}C_{t}^{*}+TB_{t}^{*},$$

where $I_{t}^{*}$ is private investment, and $TB_{t}^{*}$ are nominal net exports (including commodity exports).

## Appendix B Data, calibration, and posterior estimates

### B1 Data sources

The analysis uses quarterly and annual data for the period 1998q4 to 2021q4, based on the data set of the European Commission’s Global Multi-country Model (Albonico et al., 2019). This appendix repeats the description contained therein for convenience. Data for EMU countries and the Euro Area aggregate (EA19) are taken from Eurostat (in particular, from the European System of National Account ESA95). Bilateral trade flows are based on trade shares from the GTAP trade matrices for trade in goods and services. The Rest of the World (RoW) data are annual data and are constructed using IMF International Financial Statistics (IFS) and World Economic Outlook (WEO) databases.

Series for GDP and prices in the RoW start in 1999 and are constructed on the basis of data for the following 59 countries: Albania, Algeria, Argentina, Armenia, Australia, Azerbaijan, Belarus, Brazil, Bulgaria, Canada, Chile, China, Colombia, Croatia, Czech Republic, Denmark, Egypt, Georgia, Hong Kong, Hungary, Iceland, India, Indonesia, Iran, Israel, Japan, Jordan, Korea, Lebanon, Libya, FYR Macedonia, Malaysia, Mexico, Moldova, Montenegro, Morocco, New Zealand, Nigeria, Norway, Philippines, Poland, Romania, Russia, Saudi Arabia, Serbia, Singapore, South Africa, Sweden, Switzerland, Syria, Taiwan, Thailand, Tunisia, Turkey, Ukraine, United Arab Emirates, United Kingdom, USA and Venezuela. When not available, quarterly-frequency data are obtained by interpolating annual data using the TRAMO-SEATS package developed by Gómez and Maravall (1996).

Table B.1 lists the observed time series. GDP deflators and relative prices of aggregates are computed as the ratios of current price value to chained indexed volume. The trend component of total factor productivity is computed using the DMM package developed by Fiorentini et al. (2012). The obtained series at quarterly frequency is then used to estimate potential output.

We make a few transformations to the raw investment series. In particular, we compute the deflator of public investments based on annual data and then obtain its quarterly frequency counterpart through interpolation. This series, together with nominal public investment, is then used to compute real quarterly public investments. In order to assure consistency between nominal GDP and the sum of the nominal components of aggregate demand, we impute change in inventories to the series of investments.

### B2 Calibration and posterior estimates

Tables B.2 and B.3 reports calibrated parameter values and estimated shock processes, respectively.

## Appendix C Additional results

Sections C.1 and C.2 provide additional results on model fit and parameter stability, respectively.

### C1 Model fit

Tables C.1 and C.2 report the fit of the different models considered in Section 9. The $R^{2}$ values need, of course, to be taken with a grain of salt. The goal of the model is not to predict the pandemic, but to interpret the economic turbulence plausibly once it has materialised. Tables C.1 and C.2 show that the enhanced models with COVID-specific shocks improve the prediction of output growth during the pandemic period. However, the FS model still faces some shortcomings, because it predicts a stronger recovery than observed in the data, as discussed in the main text. Moreover, note that the extreme shocks since 2020q1 strongly affect parameter estimates (see below) and model forecasts for the re-estimated model without COVID shocks. As a result, including the COVID sample in the model estimation reduces the fit of this model (shown in column “base, est. 21q4”) substantially for the *pre*-COVID period. Moreover, the inclusion of the pandemic sample reduces the fit of hours worked significantly across models.

### C2 Parameter stability

We conclude by considering that the extreme economic volatility in the data has consequences for inference. Table C.3 shows that the additional data for 2020q1-2021q4 lead to substantial changes in the parameter estimates.^34^ Notably, without COVID-specific shocks, the model cannot reconcile the massive fluctuations in private consumption with the strong habit persistence identified in the estimation until 2019. As a result, the parameter estimate for habits drops from 0.88 to 0.19. Similarly, the discount factor shock is less persistent with an AR(1) coefficient of 0.4. Conversely, the model versions with COVID-specific shocks preserve more of the parameter estimates related to endogenous and exogenous consumption dynamics. In particular, the point estimate of habits remains very close to the pre-COVID estimation. The model versions with COVID-specific shocks also maintain better stability of the real wage rigidity parameter. The changes in the labour supply elasticity and the employment (hours) adjustment costs, however, suggest potential gains from more detailed modelling of labour hoarding and labour demand. These extensions could also improve the fit of hours worked (see Section C.1).

## Footnotes

1 Outside the macro-modelling literature, Balleer et al. (2020) present evidence from German firm-level survey data for the COVID-19 period that show supply and demand forces to coexist, but that also point to demand shortages as the main driver in the short run. In particular, the reported negative impact of COVID-19 on current business is associated with a rise in the probability of reducing prices in the replies. Sheridan et al. (2020) analyse real-time transaction data for Denmark and Sweden that point to the importance of voluntary restrictions as opposed to social distancing laws in consumption behaviour. They exploit that Denmark and Sweden were similarly exposed to the pandemic, but only Denmark mandated significant legal restrictions on social and economic activities.

2 With the benefit of hindsight, one may argue that early combinations of epidemiology and macro models have been too optimistic, or too deterministic, in the context of COVID-19 by abstracting from the proliferation of variants of the virus and the (related) re-infection risk for recovered and vaccinated households.

3 We build on the European Commission’s GM model (Albonico, Calais, Cardani, Croitorov, Ferroni, Giovannini, Hohberger, Pataracchia, Pericoli, Pfeiffer, Raciborski, Ratto, Roeger, Vogel, 2019, Kollmann, Pataracchia, Raciborski, Ratto, Roeger, Vogel, 2016), which shares elements with the European Commission’s earlier macroeconomic model suite QUEST (Burgert, Roeger, Varga, in ’t Veld, Vogel, 2020, Ratto, Roeger, in’t Veld, 2009). The description here abstracts from linear taxes and introduces only the main exogenous shocks. Appendix A provides a complete account.

4 Formally, we assume that $s_{t}^{IS}=s^{IS}\exp\left(\varepsilon_{t}^{IS}\right)$, where $s^{IS}$ is the steady-state share of commodity inputs and $\varepsilon_{t}^{IS}$ is an exogenous process.

5 We measure E as the price of foreign currency in terms of domestic currency, i.e. the amount of euro per unit of RoW currency.

6 $s_{t}^{M,Z}=s^{M,Z}\exp\left(\varepsilon_{t}^{M,Z}\right)$, where $s^{M,Z}$ denotes the steady-state import share of Z.

7 $\xi_{t+1}/\xi_{t}=\exp\left(\varepsilon_{t}^{C}\right)$ implies that the Euler equations feature the time t shock process $\varepsilon_{t}^{C}$. $\varepsilon_{t}^{C}$ comprises two components, i.e. a highly persistent preference shock, capturing ‘secular stagnation’ trends, and a less persistent one, capturing more cyclical shifts in private savings. While the persistent component helps fit inflation before the pandemic, our results are robust to excluding this shock.

8 To ensure a balanced growth path, labour disutility includes a multiplicative term $C_{t}^{1-\theta }$, such that $\omega_{t}^{N}=\omega^{N}\exp\left(\varepsilon_{t}^{U}\right)C_{t}^{1-\theta }$ where $\varepsilon_{t}^{U}$ is exogenous. Similarly, we scale asset utility by marginal utility ${\bar{\lambda }}_{t}^{s}$.

9 Given their large estimated contribution to the pandemic recession, we discuss the ‘forced savings’ shock and its macroeconomic transmission in more detail in Section 6.1. Section 7 reports two estimation variants that support the deterministic heteroskedasticity assumption for ‘forced savings’, i.e. a version estimated on pre-COVID data finds no role for the shock, and a version estimated with small prior for the shock variance still indicates large ‘forced savings’ shocks in 2020-21.

10 Like, e.g., Benigno (2009) and Ratto et al. (2009), we assume that only the RoW bond is traded internationally.

11 We follow Krishnamurthy and Vissing-Jorgensen (2012), who incorporate bonds in the utility function. Other estimated macroeconomic models use similar shocks. See, e.g., Christiano et al. (2015), Gust et al. (2017), and Del Negro et al. (2017) for closed economy models. We extend the approach to government bonds, international bonds, and corporate equity. Households face a small quadratic adjustment cost for foreign bonds, which ensures stationarity of the net foreign asset position and is rebated through lump-sum payments.

12 $\varepsilon_{t}^{bw}$ distorts the first-order condition for the foreign bond and amounts to a disturbance to the uncovered interest parity (UIP) condition. Financial shocks also capture the precautionary saving behaviour of households in the absence of high-order risk. $\varepsilon_{t}^{S}$ is an investment-specific risk premium shock.

13 Quarterly annualised inflation is defined as $\pi_{t}^{C,QA}=\log(\sum_{r=0}^{3}P_{t-r}^{C})-\log(\sum_{r=4}^{7}P_{t-r}^{C})$.

14 The different taxes are omitted from the model sketch in Section 4 for simplicity, but properly included in the detailed description of the model in Appendix A.

15 See Eqs. (12), (13), (14), and (15).

16 We include a pre-sample of five quarters.

17 By interpolating annual data, we obtain quarterly-frequency RoW data (for GDP, prices, investment to GDP, and the short-term nominal interest rate). The data for RoW short-term nominal interest rates is available only until 2020 (included).

18 The estimations use the DYNARE software (Adjemian et al., 2011).

19 The large pandemic shocks pose challenges for perturbation methods with approximations around the steady state. To explore the relevance of approximation error, we have run perfect-foresight simulations with the fully non-linear model and the historical shocks identified in the model estimation. Compared to the piecewise-linear model with ‘forced savings’, differences in the shock transmission are relatively small (up to 5% of the real GDP effect at the trough of the real GDP response) and most visible for private investment. Note, however, that uncertainty and precautionary savings, which are absent in the perfect-foresight environment, could give rise to further amplification.

20 We have also tested a model version with endogenous model-consistent expectations about the ELB duration. Convergence problems arise when we extend the model without COVID-specific shocks to the COVID-19 period. Convergence is achieved for the pre-COVID model restricted to pre-COVID data and for the extended model with COVID-19 shocks that covers the entire sample. Comparing the model variants with endogenous expectations about the duration of the ELB to those with exogenous expected duration of the ELB, once at the ELB, shows the robustness of our results to the simplifying assumption that ensures convergence of all model variants tested in this paper.

21 Appendix B provides the corresponding values.

22 The calibration of $\zeta_{0}$ and $a_{0}$ follows endogenously from the steady-state relations based on Eqs. (9) and (10). At first-order, parameter $a_{1}$ does not affect model dynamics.

23 As mentioned earlier, we are agnostic as to whether ‘forced savings’ are fundamentally demand (‘scared consumers’) or supply (mandated closures) disturbances. The co-movement between output and prices as the main difference between demand (positive correlation) versus supply (negative one) shocks is weak with transitory shocks and price stickiness. If output in a sector drops to zero, the output price is even no longer observed. Figure 2 shows that the transitory savings shock has only minimal effects on inflation. Contrary to persistent savings shocks, non-persistent shocks do not affect inflation expectations over the medium term, which are an important determinant of actual inflation in the New Keynesian Phillips curve.

24 In principle, differences between the two series may reflect not only forecast error, but also revised historical data. However, revisions of historical data have been minor (practically negligible) compared to the size of the forecast error for 2020-21. The model-based decomposition takes account of forecast errors for the large set of variables included in the Commission forecasts, i.e. not only GDP growth.

25 An example is the investment risk shock, which describes a wedge between actual investment and the investment demand compatible with average financing costs and expected future returns to investment. The wedge can have different interpretations, e.g. risk aversion, an increase in corporate financing costs over safe interest rates, or quantitative restrictions on credit volumes. Kollmann et al. (2016) illustrate the close co-movement between estimated EA and US investment risk shocks and indicators of credit tightness during 2000–14.

26 The marginal likelihood drops massively for the model without COVID-specific shocks estimated until 2019Q4 when applied to the sample until 2021Q4. This deterioration results from the large weight of the pandemic data in the marginal likelihood computation. In particular, the pre-COVID estimation implies a small prediction variance, which scales the importance of the significant forecast errors in 2020 and 2021. Therefore, the model-implied probability distribution is in contradiction to the extreme fluctuations in the COVID-19 period.

27 A generalised specification, which allows each shock type to occur throughout 2020 and 2021, produced similar results. For a sharper contrast with our benchmark model, the results focus on the ‘pre-determined’ wave-specific shocks.
